# 1α,25(OH)_2_D_3_ reverses exhaustion and enhances antitumor immunity of human cytotoxic T cells

**DOI:** 10.1136/jitc-2021-003477

**Published:** 2022-03-22

**Authors:** Peng Li, Xinhai Zhu, Guangchao Cao, Ruan Wu, Ke Li, Wenhui Yuan, Biyun Chen, Guodong Sun, Xichun Xia, Hua Zhang, Xiao Wang, Zhinan Yin, Ligong Lu, Yunfei Gao

**Affiliations:** 1 Guangdong Provincial Key Laboratory of Tumor Interventional Diagnosis and Treatment, Zhuhai Institute of Translational Medicine, Zhuhai People's Hospital Affiliated with Jinan University, Jinan University, Zhuhai, Guangdong, China; 2 The Biomedical Translational Research Institute, Faculty of Medical Science, Jinan University, Guangzhou, Guangdong, China; 3 Department of Oncology, First Affiliated Hospital, Jinan University, Jinan University, Guangzhou, Guangdong, China; 4 Anhui Provincial Center for Disease Control and Prevention, Hefei, Anhui, China; 5 Department of Infectious Disease, Guangdong Second Provincial General Hospital, Guangzhou, Guangdong, China; 6 Sun Yat-sen University Cancer Center, Guangzhou, Guangdong, China; 7 Department of Orthopedics, First Affiliated Hospital, Jinan University, Guangzhou, Guangdong, China

**Keywords:** translational medical research, immunotherapy, immunity, innate, costimulatory and inhibitory T-cell receptors

## Abstract

**Background:**

Epidemiological surveys have revealed that low serum vitamin D level was correlated with increased risk of tumors. Dysfunctional T cells in patients with tumor are characterized as exhausted with high levels of immune checkpoint receptors (ICRs). However, whether the reduced level of vitamin D in patients with cancer correlates with cytotoxic T-cell exhaustion is unknown.

**Methods:**

Periphery blood samples from 172 patients with non-small cell lung cancer (NSCLC) were prospectively collected. Patients with NSCLC received one course of intravenous docetaxel (75 mg/m^2^) followed by treatment with or without rocaltrol at a dose of 0.5–2.0 µg/day for total of 3 weeks. We performed phenotypical and functional analysis of T-cell through flow cytometry. Vitamin D receptor (VDR) knockout and overexpression CD8^+^ and Vδ2^+^ T cells were constructed using Cas9-gRNA targeted and overexpressing approaches to identify 1α,25(OH)_2_D_3_/VDR-mediated transcription regulation for ICRs or antitumor activity in T cells.

**Results:**

We show that serum level of vitamin D is negatively correlated with expression of programmed cell death-1 (PD-1), T-cell immunoreceptor with Ig and ITIM domains (TIGIT), and T-cell immunoglobulin and mucin-domain containing-3 (Tim-3), but positively correlated with CD28 expression on CD8^+^ and Vγ9Vδ2^+^ T cells in patients with NSCLC. 1α,25(OH)_2_D_3_, the active form of vitamin D, promotes the nuclear translocation of VDR, which binds to the promoter region of *Pdcd1*, *Tim3*, and *Tigit* genes and inhibits their expression. Besides, 1α,25(OH)_2_D_3_ pretreatment also promotes the methylation of CpG island in the promoter region of the *Pdcd1* gene and increases H3K27 acetylation at the promoter region of the *Cd28* gene, which leads to surface PD-1 downregulation and CD28 upregulation, respectively. We further reveal that VDR-mediated Ca^2+^ influx enhanced expression of Th1 cytokines via T-cell receptor activation. Functionally, 1α,25(OH)_2_D_3_ pretreated CD8^+^ T cells or Vγ9Vδ2^+^ T cells showed increased Th1 cytokine production and enhanced antitumor immunity. Finally, oral 1α,25(OH)_2_D_3_ could also decrease expression of PD-1, Tim-3, TIGIT and increase expression of CD28, resulting in cytokine production (associated with antitumor immunity) by cytotoxic T cells of patients with NSCLC.

**Conclusions:**

Our findings uncover the pleiotropic effects of 1α,25(OH)_2_D_3_ in rescuing the exhausted phenotype of human cytotoxic T cells in patients with tumor and in promoting their antitumor immunity.

**Trial registration number:**

ChiCTR2100051135.

## Introduction

Cancer is a global threat to human health. There is an estimated 19.3 million new cancer cases and 10 million cancer deaths in 2020.^1^ Tumor tissues are infiltrated with multiple types of immune cells which contain cytotoxic T cells such as γδ T cells^2^ and CD8 T cells^3^ and provide one of the major protections in antitumor immunity. γδ T cells recognize danger signals associated with tumorigenesis and are major players in tumor immune surveillance.^2 4 5^ Besides, γδ T cells provide the early source of interferon gamma (IFN-γ) in tumor immunity and have potent cytotoxic property.^6^ These cells recognize antigen in a major histocompatibility complex (MHC)-independent manner,^7^ and clinical trials revealed promising effects in autogenic and allogeneic γδ T-cell therapy.^8–10^ CD8 T cells are antigen-specific tumor killers and are the most important cytotoxic T cells in many cancers.^3^ Currently, most tumor immune therapy strategies, such as chimeric antigen receptor T-cell therapy (CAR-T), are based mainly on αβ T cells (CD4 and CD8) and have achieved exciting success in clinic.^3 11^

However, tumor cells adopt many approaches to inhibit antitumor immunity mediated by cytotoxic T cells.^12–14^ Cytokines, such as IFN-γ, stimulate expression of programmed cell death 1-ligand 1 (PD-L1) on tumor cells, which binds to programmed cell death-1 (PD-1) on cytotoxic cells and reduce their effector function.^15^ The binding of T-cell immunoglobulin and mucin-domain containing-3 (Tim-3) with galectin-9 (Gal-9) or carcinoembryonic antigen cell adhesion molecule 1 (CEACAM-1) in tumor microenvironment negatively regulates antitumor response.^16 17^ Tumor or dendritic cells express poliovirus receptor, which binds to T-cell immunoreceptor with Ig and ITIM domains (TIGIT) on CD8 T cells and suppresses their tumor killing ability.^18^ Besides, the B7/CD28 costimulatory signaling pathway is also a necessity for antitumor immune response, but tumor burden reduces CD28 expression on cytotoxic T cells.^19^ CTLA4/LAG3/FAS-mediated inhibitory signals also hinder anti-tumor responses.^12 13^ These inhibitory effects converged to induce exhaustion of cytotoxic T cells and dampen antitumor immunity. Animal models and clinical studies have shown that Tim-3, TIGIT, LAG-3, PD-1, and PD-L1 targeted therapies have promising effects in enhancing antitumor immunity in a wide variety of tumor types.

Vitamin D is most widely known for its beneficial effects on calcium homeostasis and bone mineralization.^20^ After being taken up from diet or synthesized by the skin, vitamin D is hydroxylated at C25 by CYP2R1 in the liver to 25(OH)D_3_; the latter is further hydroxylated at C1 by CYP27B1 into 1α,25(OH)_2_D_3_ (calcitriol), the bioactive form.^21^ 1α,25(OH)_2_D_3_ binds to vitamin D receptor (VDR), which functions as a transcription factor to regulate various biological processes.^22^ Recent studies revealed that vitamin D also has important regulatory functions on the immune system: vitamin D not only enhances pathogen clearance mediated by innate cells but also exerts immunosuppressive effects to prevent the detrimental responses of prolonged inflammation.^23^ Epidemiological surveys have revealed an association between low serum 25(OH)D_3_ levels and increased risk of prostate, lung, coloretcal, breast, and hepatic cancers.^24–26^ The antiproliferative effects of vitamin D on tumor cells were also demonstrated,^27^ but whether vitamin D regulates antitumor immunity mediated by cytotoxic T cells is barely known.

Here, we showed that the expressions of PD-1, Tim-3 and TIGIT on Vγ9Vδ2^+^ T cells and CD8^+^ T cells are regulated by the 1α,25(OH)_2_D_3_/VDR signaling pathway, which contributes to T-cell cytotoxicity under the condition of tumor-antigen and T-cell receptor (TCR) activation. 1α,25(OH)_2_D_3_ also modulates the epigenetic programs of *Pdcd1* and *Cd28* genes, which results in subsequent reduced expression of PD-1 but upregulation of CD28. Besides, we preliminarily reveal that the production of Th1 cytokines in T cells is dependent on VDR and Ca^2+^ influx. Mouse models reveal that Vγ9Vδ2^+^ T cells pretreated with 1α,25(OH)_2_D_3_ exert impressive antitumor activity in vivo. In a clinical trial, patients with cancer with NSCLC who received oral calcitriol (1α,25(OH)_2_D_3_) showed decreased cell-surface level of PD-1, Tim-3 and TIGIT and increased CD28 expression on Vγ9Vδ2^+^ and CD8^+^ T cells. Peripheral blood T lymphocytes isolated from 1α,25(OH)_2_D_3_-treated patients with NSCLC showed increased expression of cytokines associated with antitumor immunity. These results converged to strongly support a promotive effect of 1α,25(OH)_2_D_3_/VDR on T-cell cytotoxicity and reveal potential targets for immunotherapy.

## Materials and methods

### Mice

Wild-type (WT) mice and NOD.CB17-Prkdc^scid^/NcrCrl (NOD-SCID) mice were purchased from Beijing Vital River Laboratory Animal Technology Co. All animals were maintained in a specific pathogen-free facility for use according to the guidelines for experimental animals at Jinan University (Guangzhou, China). Mice were used between 5 weeks and 8 weeks of age.

### Expansion of Vγ9Vδ2 T cells

Blood from healthy male or female donors was obtained from the Guangzhou Blood Center. Human peripheral blood mononuclear cells (PBMCs) were isolated by Ficoll-Paque-based (GE Healthcare, 17-1140-02) density gradient centrifugation. Zoledronate (ZOL) (Sigma, SML0223) expanded Vγ9Vδ2 T (Vδ2 T) cells were generated as described following.^28^ Briefly, human PBMCs (2–3×10^6^/mL) were cultured in RPMI 1640 (Gibco, 11875093) medium supplemented with 10% Fetal Bovine Serum (FBS, Gibco, 16140071). Recombinant human interleukin (IL)-2 (Peprotech, 200-02-50) 400 U/mL and ZOL (50 µM) was added at day 0. Recombinant human IL-2 was added to a final concentration of 100 U/mL once every 2 days from day 3. T cells were maintained at a cell density of 1–2×10^6^/mL until the percentage of Vγ9Vδ2/CD3 T is >90%. The purity of Vγ9Vδ2 T cells was determined by flow cytometry (BD VERSE). After 10–12 days of culture, cells were used in experiments. In some experiments, the Vγ9Vδ2 T cells were further purified by negative selection with EasySep Human Gamma/Delta T Cell Isolation Kit (STEM CELL, 19255). Unless mentioned otherwise, Vγ9Vδ2 T cells used in all the experiments were pretreated with 1α,25(OH)_2_D_3_ (50 nM) or vehicle two times at 1-day intervals.

### CD3^+^, CD4^+^, and CD8^+^ T-cell isolation

Human CD3^+^, CD4^+^, and CD8^+^ T cells were isolated with magnetic microbeads (BD, 552593, 557766, and 557767), respectively. The purity of the enriched subset was validated by flow cytometry and was generally higher than 95% (CD3^+^, CD4^+^, and CD8^+^/CD3 T cells). Cells were activated with plate bound anti-human CD3 (5 µg/mL; BioLegend, 300314) and anti-human CD28 (1 µg/mL; BioLegend, 302923) for 48 hours in the presence of 400 U/mL IL-2. The medium was refreshed on day 2. CD3^+^, CD4^+^, and CD8^+^ T cells were cultured in RPMI-1640 medium and supplemented with 100 U/mL IL-2, and 10% FBS (Gibco, 10 270–106). Unless mentioned otherwise, CD3^+^, CD4^+^, and CD8^+^ T cells used in all the experiments were pretreated with 1α,25(OH)_2_D_3_ (50 nM) or vehicle two times at 1-day intervals.

### Inhibition of methyltransferase and deacetylase in T cells

For methyltransferase inhibition, Vγ9Vδ2^+^ or CD8^+^ T cells were treated with 5′-aza-2′-deoxycytidine (1 µM; Sigma, A3656), 1α,25(OH)_2_D_3_ (50 nM; Enzo, BML-DM200-0050) or the combination for 48 hours. In some experiments, the frequency of DNA methylation at the CpG islands in *Pdcd-1* promoter regions was detected by pyrosequencing (Beijing Genomics Institute BGI). For histone deacetylase inhibition, Vγ9Vδ2^+^ or CD8^+^ T cells were treated with trichostatin (100 nM, trichostatin A (TSA); Selleck, S1045), 1α,25(OH)_2_D_3_ (50 nM), or the combination for 48 hours.

The clinical trial was registered in Chinese Clinical Trial Registry.

### Human subjects

A total of 172 patients with NSCLC were recruited from the Department of Oncology, First Affiliated Hospital of Jinan University. A total of 47 healthy donors were recruited from the staff at the Jinan University. Information of patients with cancer and healthy donors is described in online supplemental tables 1 and 2. PBMCs were isolated by Ficoll-Paque-based density gradient centrifugation or stored at −80℃. Cell-surface levels of exhaustion markers PD-1, Tim-3, TIGIT, and costimulatory molecule CD28 on CD4^+^, CD8^+^, and Vγ9Vδ2^+^ T cells were determined by flow cytometry.

10.1136/jitc-2021-003477.supp1 Supplementary data

### In vitro treatment of T cells

To measure cell-surface levels of PD-1, Tim-3, TIGIT, and CD28 in human CD8^+^ and Vγ9Vδ2^+^ T cells, PBMCs were isolated from patients with NSCLC by gradient centrifugation. PBMCs were then stimulated with 1α,25(OH)_2_D_3_ (50 nM) three times at 2-day intervals in vitro. In some experiments, PBMCs were isolated from healthy donors by gradient centrifugation. Purified CD8^+^ and Vγ9Vδ2^+^ T cells were treated with 1α,25(OH)_2_D_3_ (50 nM) three times at 2-day intervals and then were harvested for further analysis.

### Study design

After obtaining informed consent, therapy cycles were scheduled according to the clinical trial standard, approved drug interval and health condition of each patient. For rocaltrol treatment, all patients with NSCLC received one course of intravenous docetaxel (75 mg/m^2^) followed by rocaltrol at a dose of 0.5–2.0 µg/day for a total of 3 weeks. For the control group, patients with NSCLC received one course of intravenous docetaxel (75 mg/m^2^) without rocaltrol for 3 weeks. Briefly, blood samples of patients were harvested at baseline (before therapy initiation). Patients with NSCLC were administrated docetaxel (75 mg/m^2^) or docetaxel combined with rocaltrol (0.5–2.0 µg/day, Roche) for a total of 3 weeks (online supplemental table 2). Blood samples were collected from the patients after 3 weeks of therapy. Fresh trial serum and PBMCs were detected by ELISA and flow cytometry.

### Animal model

In breast cancer model, NOD-SCID mice were inoculated subcutaneously with 5×10^6^ MCF-7 cells. Ten days later, when MCF-7 tumors had reached a volume of 50 mm^3^, the mice were randomized into three groups and treated respectively with Vγ9Vδ2 T cells (10×10^6^), 1α,25(OH)_2_D_3_ pretreated Vγ9Vδ2 T cells (10×10^6^), 100 µL Phosphate Buffer Solution (PBS) as control. All animals were transferred intravenously with Vγ9Vδ2 T cells or PBS once a week.

In a diffuse large B-cell lymphoma (DLBCL) tumor model, NOD-SCID mice were inoculated subcutaneously with 10×10^6^ U2932 cells. One week later, when U2932 tumors had reached a volume of 50–100 mm^3^, the mice were randomized into six groups and were treated respectively with 1α,25(OH)_2_D_3_−Vγ9Vδ2 T cells, vehicle−Vγ9Vδ2 T cells, 1α,25(OH)_2_D_3_−Vγ9Vδ2 T cells+αPD-L1, vehicle−Vγ9Vδ2 T cells+αPD-L1, ibrutinib, and PBS. Vγ9Vδ2 T cells (10×10^6^) and Vγ9Vδ2 T cells pretreated with 1α,25(OH)_2_D_3_ (10×10^6^) were intravenously injected into mice once every 5 days. αPD-L1 antibody (250 µg, atezolizumab) was intravenously injected every 3 days (three times in total), 7 days after U2932 inoculation. For positive control, mice received ibrutinib (25 mg/kg, MCE, HY-10997) once every 2 days from days 2 to 22. Vγ9Vδ2 T cells were treated with 1α,25(OH)_2_D_3_ three times 1 day before adoptive cell-transfer therapy.

In a B16-F0 tumor model, age-matched and sex-matched B6 WT mice (age of 6–8 weeks) were inoculated subcutaneously with 4.0×10^5^ B16-F0 cells. On day 0, the mice were randomized into two groups, and PBS (200 µL) or 1α,25(OH)_2_D_3_ (0.03 mg/kg, 200 µL), were intravenously injected into the mice every 2 days, and eight times in total. Tumor size and mice survival were recorded every 2 days from day 6. Tumor volume (TV) was recorded and calculated using the following equation:

$$TV=\frac{L\times W^{2}}{2}$$

where W=width, L=length. Mice bearing a tumor with size larger than 15 mm in any direction were euthanized.

### Tumor-infiltrating lymphocyte (TIL) isolation

In order to analyze the cytokine production and expression of surface markers on tumor-infiltrating T cells (TILs), mice were euthanized on day 18. Tumor tissues were cut into pieces and suspended with 10 mL tumor digestion buffer (5% FBS, 1.5 mg/mL collagenase IV, and 10 µg/mL DNase I). After rotation for 1 hour at 37℃, tumor tissues were digested. The cell suspension was filtered with a 70 µm filter to harvest single-cell suspension. Leukocytes were isolated by density-gradient centrifugation using 40% and 70% Percoll (GE, 17089102). Then, tumor-infiltrating leukocytes were labeled with specific antibodies for different markers.

### Cytotoxic assay

To determine the cytotoxicity of 1α,25(OH)_2_D_3_ pretreated Vγ9Vδ2 T cells, human tumor cell lines were used to perform killing assays. Tumor cells (target, T) were prelabeled with 2.5 µM 5(6)-carboxyfluorescein diacetate succinimidyl ester (CFSE) (Thermo Fisher, 65-0850-84). 1α,25(OH)_2_D_3_ pretreated Vγ9Vδ2 T cells (effector, E) and target cells were coincubated at different effector:target ratios (0:1, 1:1, 5:1, and 10:1) at 37℃ for 6 hours. The percentages of dead cells among total target cells were identified as PI^+^ (Sungene Biotech, AO2002-H) staining with flow cytometry. Cytotoxicity was calculated based on this equation:

$$(\%killing)=\frac{Experimentalgroupdeathrate-Naturaltargetcellmortality}{100-Naturaltargetcellmortality}\times 100\%$$

### Flow cytometry

For cell-surface staining, approximately 10^5^–10^6^ human Vγ9Vδ2^+^ T or CD8^+^ T cells were incubated with specific antibodies for 15 min at 4℃ in the dark. For intracellular staining, cells were restimulated with plate bound anti-human CD3 (5 µg/mL) and anti-human CD28 (1 µg/mL) or 50 ng/mL phorbol 12-myristate 13-acetate (PMA, Sigma, P8139) and 1 µg/mL ionomycin (Sigma, I9657) in the presence of Golgi Stop (1:1000 dilution; BD Biosciences, 554724) for 4 hours. In some experiments, TILs were stimulated with 5 µg/mL anti-mouse CD3 (BioLegend, 300438) and 1 µg/mL anti-mouse CD28 (BioLegend, 102116) in the presence of Golgi Stop for 4 hours, then fixed and permeabilized with BD Cytofix/Cytoperm Plus (BD Biosciences, 554715), and incubated with specific antibodies for another 30 min at 4℃ in the dark, all procedures were performed according to the manufacturer’s recommendations (BD Biosciences, 554715). All samples were acquired with FACS Verse flow cytometer (BD Biosciences) and analyzed with FlowJo software (TreeStar). The following antibodies were used: PerCP-conjugated anti-human TCR Vδ2 (BioLegend, 331410), V500-conjugated anti-human CD3 (BD Biosciences, 561416), PE-conjugated anti-human CD28 (BioLegend, 302907), Pacific Blue-conjugated anti-human CD279 (BioLegend, 329915), PerCP-conjugated anti-human CD8 (BioLegend, 300921), PerCP-conjugated anti-human CD4 (BioLegend, 317431), APC-conjugated anti-human tumor necrosis factor alpha (TNF-α) (BioLegend, 502913), PE/Cy7-conjugated anti-human perforin (BioLegend, 353315), Pacific Blue-conjugated anti-human/mouse granzyme B (BioLegend, 515407), Brilliant Violet 421-conjugated anti-human CD95 (BioLegend, 305623), APC-conjugated anti-human CD107a (BioLegend, 328620), fluorescein isothiocyanate (FITC)-conjugated anti-human IFN-γ (BioLegend, 506504), PE-conjugated anti-human CTLA-4 (BioLegend, 369603), PE/Cy7-conjugated anti-human NKG2D (BioLegend, 320811), APC-conjugated anti-human Tim-3 (BioLegend, 345011), PE/Cy7-conjugated anti-human TIGIT (BioLegend, 372714), Brilliant Violet 421-conjugated anti-human Ki-67 (BD Biosciences, 562899), APC conjugated anti-mouse IFN-γ (BioLegend, 505809), FITC conjugated anti-mouse TNF-α (BioLegend, 506303), PE/Cy7 conjugated anti-mouse CD3 (BioLegend, 100219), PE conjugated anti-mouse CD4 (BioLegend, 130310), PerCP/Cyanine5.5 conjugated anti-mouse CD8 (BioLegend, 140417), and Brilliant Violet 421 conjugated anti-mouse TCR γ/δ (BioLegend, 118119).

### CRISPR/Cas9-mediated gene disruption and overexpression

Three short guide RNAs (sgRNAs) for per gene were designed using the online tool provided by the Zhang laboratory (MIT, http://tools.genome-engineering.org). sgRNA sequences were in online supplemental table 3. Oligonucleotide pairs with BsmBI-compatible overhangs were annealed and cloned into the lentiviral vector lenti-CRISPR v2 (Addgene plasmid, 52961). For virus production, HEK293T cells were transfected with lenti-CRISPR V.2, psPAX2 (Addgene, 12260) and pMD2.G (Addgene plasmid, 12259) at a 10:5:1 ratio using Lipofectamine 3000 transfection reagent (Invitrogen, L3000015) and cultured in RPMI-1640 with 10% fetal bovine serum. The medium was replaced 12 hours after transfection and virus were harvested from the supernatant after a further 48–56 hours. Cell debris was removed by centrifugation (10 min at 2000 rpm, 4℃). Virus-containing suspension was concentrated overnight (12–16 hours) with virus concentration kit (Clontech, 631231). For lentivirus transduction, purified CD8^+^ or Vγ9Vδ2^+^ T cells were transduced with lentivirus carrying CRISPR-Cas9-*VDR* and their control CRISPR-Cas9-NC, respectively, at 500×*g* for 90 min at 4℃. Subsequently, cells were cultured in normal medium for an additional 2 days. After puromycin selection two times, T cells were harvested and detected by western blot and quantitative real-time PCR. Furthermore, transduced T cells were restimulated with 1α,25(OH)_2_D_3_ (50 nM) two times at 1-day intervals and determined by flow cytometry. For overexpression *VDR*, CD8^+^ T cells were transfected with PTSB–CMV–GFP–*VDR* (The *VDR* overexpression gene was inserted into a CMV expression vector with a GFP gene) and control vector PTSB–CMV–GFP (TransSheep Biotechnology), respectively, at 500×*g* for 90 min at 4℃. Cells were then restimulated with 1α,25(OH)_2_D_3_ (50 nM) two times at 1-day intervals. The cell-surface levels of immune checkpoint receptors (ICRs) were detected by flow cytometry. The decline ratio of ICRs was calculated according to following formula:

Ratio(%)=PTSB−CMV−NC/VDR(−1.25D3)−PTSB−CMV−NC/VDR(+1.25D3)PTSB−CMV−NC/VDR(−1.25D3)

### RNA extraction, cDNA synthesis, and quantitative real-time PCR

Cells were harvested in 1 mL TRIzol (Invitrogen, Thermo Fisher Scientific), and total RNA was isolated with RNA simple Total RNA kit (Tiangen, DP419). RNA from each sample (200–500 ng) was reversely transcribed with the PrimeScript RT reagent kit in accordance with the manufacturer’s instructions (TaKaRa, RRO37A). The cDNA was diluted at 1:5 or 1:10 in RNase/DNAse-free water for further analysis of quantitative real-time PCR (qPCR). Expression of target genes was determined by CFX Connect System (Bio-Rad) with 2×SYBR Green PCR Master Mix (Bimake, B21203) according to the manufacturer’s instructions, and each qPCR reaction had a final volume of 20 µL. β-actin or GAPDH was used as the internal control. Each sample was examined at least in triplicate. PCR product specificity was confirmed by a melting-curve analysis. The relative mRNA expression was calculated using 2^−ΔΔCt^ method. Hot map was described as fold change according to the following formula:

$$Foldchange=log_{2}^{2^{-(\Delta Ct_{target}-\Delta Ct_{reference})}}$$

Primer pairs for quantitative real-time PCR were purchased from Sangon Biotech. The list of primer sequences is provided in online supplemental table S4.

### Pyrosequencing

The PyroMark Assay Design V.2.0 software was used to design primers for the analysis of iNOS methylation. Briefly, the following primer sequences were used: 5′-(Btn)- −3′ (5-biotin labeled) and 5′- -3′, sequencing primer: bisulfite conversion was carried out using the EpiTect Plus DNA Bisulfite Kit (QIAGEN). We carried out the bisulfite conversion following the manufacturer’s guidelines (QIAGEN). The PCR amplification was conducted in 50 µL reactions containing 2 µL of DNA (bisulfite conversion), 1 µL PCR primers, 2 µL dNTP mix, 10 µL 5×PCR GC buffer, and 0.2 µL 5 U Taq DNA polymerase (KAPA Biosystems). The PCR conditions were as following: 95°C for 3 min, 45 cycles of (94°C for 30 s, 50°C for 30 s and 72°C for 1 min) followed by 7 min hold at 72°C and then 4°C. The PCR products were then captured using streptavidin coated agarose beads under denaturing conditions to obtain single-stranded DNA. The pyrosequencing reaction was then carried out using the PyroMark Q96 machine (QIAGEN) and PyroMark Gold-Q96 Reagents kit (QIAGEN), using the following sequencing primer: 5′- -3. The methylation status was analyzed using TPyro Q-CpG software (QIAGEN). The frequency of DNA methylation at the CpG islands in *Pdcd-1* promoter regions were detected by pyrosequencing (Beijing Genomics Institute BGI). The primer sequences are provided in online supplemental table 5.

### ChIP-qPCR and ChIP-seq

Chromatin immunoprecipitations were performed according to the protocol of Simple ChIP Enzymatic Chromatin IP Kit (CST, 9003) as previous described.^29^ For VDR binding detection, CD8^+^ T cells (5×10^6^–1×10^7^) were treated with 1α,25(OH)_2_D_3_ (50 nM) for 12 hours followed by chromatin immunoprecipitations. For acetylation detection, Vγ9Vδ2^+^ T cells (5×10^6^–1×10^7^) were treated with 1α,25(OH)_2_D_3_ (50 nM) or vehicle two times at 1-day intervals, followed by chromatin immunoprecipitations. Briefly, cells were fixed for 12 min at room temperature with 1% formaldehyde, followed by addition of glycine, and incubated for another 5 min at room temperature. Subsequently, cells were lysed; chromatin was harvested and fragmented (150–900 bp) using enzymatic digestion. The chromatin was then subjected to immunoprecipitation with specific antibodies. Rabbit polyclonal antibodies to anti-VDR (CST, 12550), anti-H3K27ac, anti-H3 (CST, 4620), and normal rabbit IgG (CST, 8173) were used. Finally, the immune complexes were washed and eluted. Eluted DNA and 2% input DNA were incubated at 60℃ to reverse the cross-linking and then purified with spin columns. The relative abundance of precipitated DNA fragments was analyzed by qPCR using SYBR Green PCR Master Mix. Primers (forward and reverse) for amplification of the region upstream of *Pdcd1*, *Cd28*, *Tigit*, and *Tim-3* are provided in online supplemental table 6. The results are showed as being relative to the total chromatin input or normalized to total histone H3 expressions to account for the nucleosomal occupancy at the promoter regions of genes. In addition, ani-H3K27ac enriched DNA fragments were used for further chromatin immunoprecipitation sequencing (Beijing Genomics Institute BGI).

### Confocal microscopy

For VDR detection, purified Vγ9Vδ2^+^ T cells were treated with 1α,25(OH)_2_D_3_ (50 nM) for 12 hours and washed once by PBS. In some experiments, vehicle or 1α,25(OH)_2_D_3_ (50 nM) pretreated Vγ9Vδ2 T cells were stimulated with anti-human CD3 (5 µg/mL) and anti-human CD28 (1 µg/mL), or PMA (50 ng/mL) plus ionomycin (1 µg/mL) at the indicated time points (0 s, 120 s, 60 min, and 4 hours). Subsequently, T cells were fixed in 4% (vol/vol) paraformaldehyde for 30 min in room temperature, cells were permeabilized with 0.2% Triton X−100 (Sigma) for 30 min, and blocked in Tris Buffered Saline (TBS) (0.02% Triton X-100% and 2% BSA in PBS) for another 2 hours. Then T cells were incubated with anti-VDR antibody (Santa Cruz Biotechnology, sc13133) at 4°C overnight. After three washes with PBS, the cells were incubated at room temperature with Alexa Fluor 488 goat anti-mouse IgG (H+L) (Thermo Fisher, CA11005S) secondary antibody (1:200 dilution) for another 2 hours. Negative controls were carried out using isotype IgG. Subsequently, the cells were counterstained with 2-(4-Amidinophenyl)-6-indolecarbamidine dihydrochloride (DAPI) for 5 min in the dark. All images were captured with a Leica TCS SP2 AOBS confocal laser scanning microscope (Leica Microsystems).

### Immunobloting analysis

CD8^+^ and Vγ9Vδ2^+^ T cells were treated with 1α,25(OH)_2_D_3_ (50 nM) three times at 1-day intervals in vitro. T cells were stimulated with anti-human CD3 (5 µg/mL) and anti-human CD28 (1 µg/mL) at the indicated time points (0 s, 30 s, 60 s, 3 min, 5 min, and 10 min). Subsequently, cells were harvested and lysed with RIPA buffer (Beyotime, P0013K), which included phosphatase inhibitor cocktail (Bimake, B15001). Total proteins (20–40 µg) were separated on 10% sodium dodecyl sulfate–polyacrylamide gel electrophoresis gels and electrotransferred to 0.22 µm PVDF membrane with the PowerPac wet-blot system (Bio-Rad). Approximately 5% BSA was used to block membranes for 1 hour, and then incubated with the indicated primary antibodies overnight at 4℃. Primary antibodies included p-Zap70 (CST, 2717), Zap70 (CST, 2705), LAT (9166), p-LAT (CST, 3584), PLCγ1 (CST, 2822), p-PLCγ1 (14008), VDR (CST, 12550), PD-1 (CST, 84651), and β-actin (CST, 3700). After six times washed in TBST for 1 hour, the membranes were incubated with HRP (Horseradish Peroxidase)-linked secondary antibody at a dilution of 1:2000 (CST, 7074) at room temperature for 2.5 hours. Finally, membranes were washed five times in TBST, and then detected by Bio-Rad ChemiDoc MP Gel imaging system (Bio-Rad).

### Calcium-flux analysis

Vγ9Vδ2^+^ T cells were pretreated with or without 1α,25(OH)_2_D_3_ (50 nM) for 2 times at 2 day intervals. In some experiments, VDR-KO Vγ9Vδ2^+^ T cells were used in following experiments. For intracellular Ca^2+^ detection, Vγ9Vδ2^+^ T cells (1×10^6^) were loaded with 2.5 µM Fluo-3 AM (Beyotime, S1056) in Ca^2+^-free medium for 40 min at 37℃, and then washed twice in Ca^2+^-free medium. Baseline measurements were achieved from the samples without stimulation for 30–60 s. At 30 or 60 s, anti-human CD3 (10 µg/mL) and anti-human CD28 (1 µg/mL), or ionomycin (1 µg/mL) plus PMA (50 ng/mL) were supplemented into cultures. T cells were harvested by flow cytometry (BD, Accuri C6) for another 360 s. In some experiments, cell cultures were supplemented with 2 mM Ca^2+^, anti-human CD3 (10 µg/mL) and anti-human CD28 (1 µg/mL), or ionomycin (1 µg/mL) plus PMA (50 ng/mL). Ca^2+^ influx indicated as fluo-3AM was analyzed by flow cytometry.

### ELISA

Supernatant of cell culture or serum was collected, 25-hydroxyvitamin D_3_ (25(OH)D_3_, mlbio) and 1α,25-Dihydroxyvitamin D_3_ (1,25(OH)_2_D_3_, Jiang lai bio, JL13863-96T, JL35250), and human cytokines were measured by precoated ELISA kits (human IFN-γ precoated ELISA kit (Dakewe, 1110002), TNF-α (Dakewe, 1117202), perforin (Dakewe, 1118302), and granzyme B (Dakewe, 1118502)), following the manufacturer’s instructions.

### Statistical analysis

Statistical analysis was performed using GraphPad Prism V.6.0 (GraphPad Software). Data are presented as the mean±SD. Statistical significance was determined as indicated in the figure legends. Significance was set to p<0.05 and represented as *p<0.05, **p<0.01, ***p<0.001, ****p<0.0001, not significant. For sample sizes of n=3, a two-tailed unpaired Student’s t-test was used when the variance was similar between two groups, and a two-tailed unpaired Student’s t-test with Welch’s correction was used when variances were different. For sample sizes of n>3, the data distribution was first checked using a Kolmogorov-Smirnov test (GraphPad Software). If the data fitted a normal distribution, a two-tailed unpaired Student’s t-test was used when variances were similar, whereas a two-tailed unpaired Student’s t-test with Welch’s correction was used when variances were different. If the data did not fit a normal distribution, a Mann–Whitney U test was used. A two-tailed paired Student’s t-test was used when the difference value was Gaussian distributed, and a two-tailed paired Student’s t-test with Wilcoxon correction was used when data were skew distribution. Correlation analysis was performed using Pearson’s test. Animals were randomly allocated to the treatment groups.

## Results

### 1α,25(OH)_2_D_3_ decreases expression of multiple ICRs but increases CD28 expression

**Figure 1:**
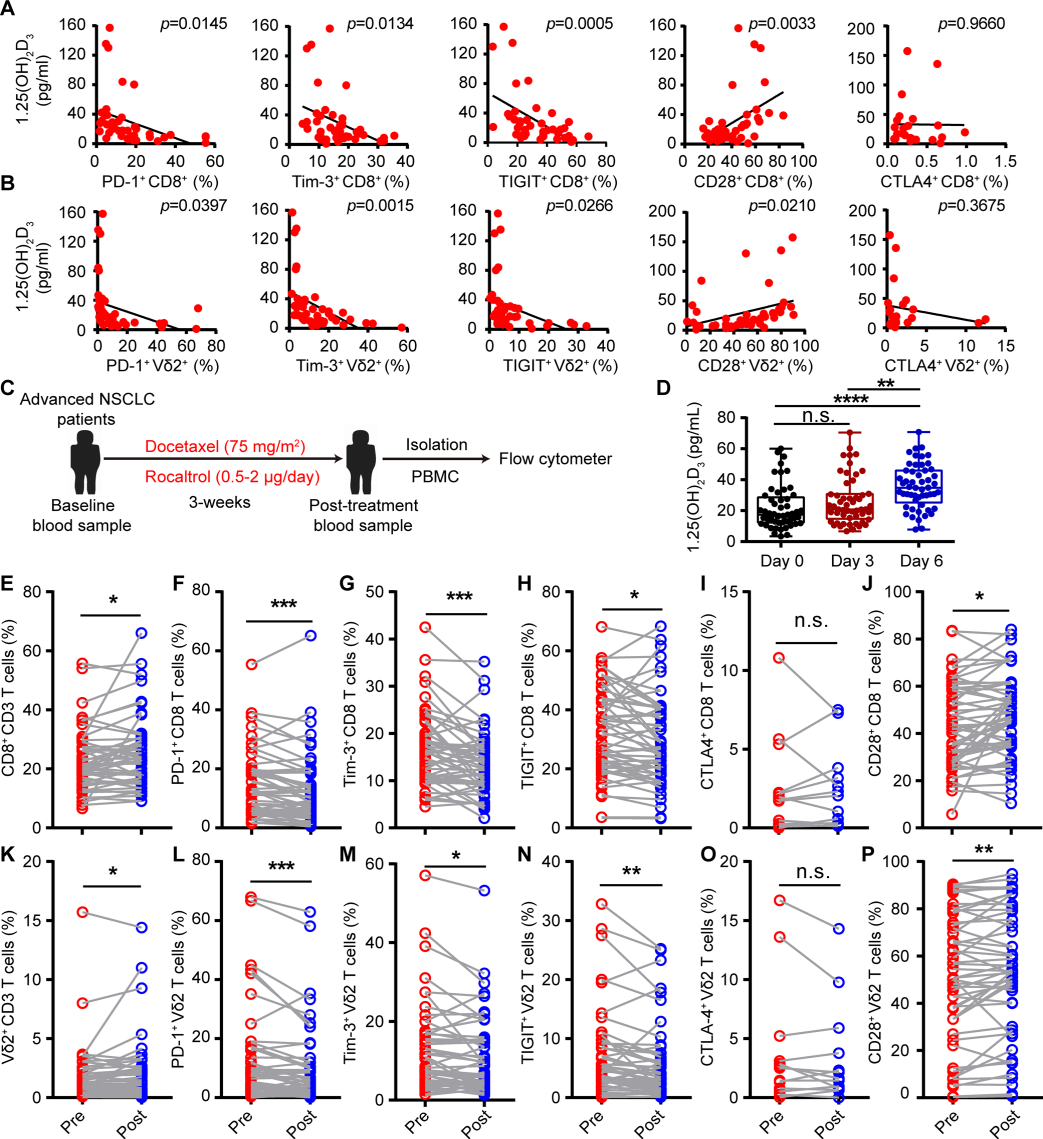
1α,25(OH)_2_D_3_ decreased expression of multiple checkpoint receptors but increased CD28 expression. (A, B) Linear regression analysis between 1α,25(OH)_2_D_3_ level and immune checkpoint receptors (PD-1^+^, Tim-3^+^, TIGIT^+^, and CTLA-4^+^) and costimulatory molecule CD28^+^ on CD8^+^, Vγ9Vδ2^+^ (Vδ2^+^) T cells derived from PBMCs of patients with advanced lung cancer. n=42 for patients with advanced lung cancer (correlation between CTLA-4^+^ expression and 1α,25(OH)_2_D_3_ level, n=23). (C) Overview of study design. Patients with NSCLC received one course of intravenous docetaxel (75 mg/m^2^) followed with rocaltrol at a dose of 0.5–2.0 µg/day for a total of 3 weeks. (D) Graphs showed the 1α,25(OH)_2_D_3_ levels in sera of patients with NSCLC, before and after therapy with rocaltrol (0.5–2.0 µg/day) at the indicated time points. n=53. (E–P), Comparison of PD-1^+^, Tim-3^+^, TIGIT^+^, CTLA-4^+^, and CD28^+^ levels on CD8^+^, Vγ9Vδ2^+^ T cells, in the peripheral blood of patients, before (pre) and after (post) treatment with rocaltrol (0.5–2.0 µg/day for a total of 3 weeks). n=52–53 (frequency of CTLA-4^+^ showing CD8^+^, Vγ9Vδ2^+^ T cells, n=13). Pearson’s correlations (A, B); unpaired Student’s t-test (D); paired Student’s t-test (F–H); Wilcoxon matched-pairs signed-rank test (E, I–P). Significance was set to p<0.05 and represented as *p<0.05, **p<0.01, ***p<0.001, ****p<0.0001. n.s., not significant; NSCLC, non-small cell lung cancer; PBMC, peripheral blood mononuclear cell; PD-1, programmed cell death-1; TIGIT, T-cell immunoreceptor with Ig and ITIM domains; Tim-3, T-cell immunoglobulin and mucin-domain containing-3.

Low-serum vitamin D level was correlated with increased risk of tumors,^24–26^ and supplementation of vitamin D reduced the risk of cancer death.^30^ Tumor burden also induces exhaustion of cytotoxic T cells and hinders antitumor immunity.^13^ However, whether reduced vitamin D participates in the etiology of exhaustion of cytotoxic T cells is unknown. Thus, we recruited 53 patients with advanced lung cancer (non-small cell lung cancer (NSCLC)), and 47 healthy volunteers, serum vitamin D (25(OH)D_3_, 1α,25(OH)_2_D_3_) levels, and baseline expression of ICRs on CD8^+^ T and Vγ9Vδ2^+^ T cells from PBMCs were analyzed. A summary of the characteristics of both healthy volunteers and patients with cancer with NSCLC is described in tables 1 and 2. Consistent with previous reports, patients with cancer showed immune exhaustion phenotype as indicated by increased cell-surface expression of PD-1, TIGIT, and Tim-3 but reduced CD28 expression on CD8^+^ T cells and Vγ9Vδ2^+^ T cells, while cell surface CTLA-4 was barely detectable (online supplemental figure S1A, B). The serum levels of vitamin 25(OH)D_3_ and the bioactive form 1α,25(OH)_2_D_3_ in patients with cancer were also significantly lower than those of healthy controls (online supplemental figure S2A, B), which was in accordance with previous studies. Importantly, we found that the serum levels of 25(OH)D_3_ and 1α,25(OH)_2_D_3_ in patients with NSCLC were negatively correlated with PD-1, TIGIT, and Tim-3, but positively correlated with the CD28 level on cytotoxic T cells (figure 1A, B, and online supplemental figure S3A, B). To test whether vitamin D could directly regulate the expression of ICRs on cytotoxic T cells, we isolated PBMC from patients with NSCLC and cultured in the presence or absence of 1α,25(OH)_2_D_3_ for 6 days (online supplemental figure S4A). Notably, 1α,25(OH)_2_D_3_ treatment significantly reduced PD-1, TIGIT, and Tim-3 but increased CD28 expression on CD8^+^ T and Vγ9Vδ2^+^ T cells (online supplemental figure S4B–D). Besides, 1α,25(OH)_2_D_3_ also significantly reduced expression of PD-1, TIGIT and Tim-3 (Co-expression of PD-1 with Tim-3 or TIGIT) and increased CD28 expression on T cells (CD8^+^, CD4^+^, and Vγ9Vδ2^+^ T cells) from healthy donors during de novo activation (online supplemental figure S5A–E). Furthermore, Vγ9Vδ2^+^ T cells pretreated with 1α,25(OH)_2_D_3_ showed higher levels of Ki-67 (online supplemental figure S5F). These results strongly indicated that vitamin 1α,25(OH)_2_D_3_ could directly regulate the expression of ICRs on T cells. We next checked whether vitamin D supplementation could reverse the exhaustion phenotype of cytotoxic T cells in patients with NSCLC. In order to do so, all patients with NSCLC were administered with one course of intravenous docetaxel (75 mg/m^2^) followed therapy with rocaltrol at a dose of 0.5–2.0 µg/day for total of 3 weeks (figure 1C). Treatment cycles were scheduled according to the clinical trial standard, approved drug interval and health condition of each patient. Patient information is provided in online supplemental table 2 (for cell-surface markers detection). After therapy initiation, patients with NSCLC showed elevated 1α,25(OH)_2_D_3_ levels in serum at the indicated time points (figure 1D). Delightedly, oral intake of calcitriol reduced PD-1, TIGIT and Tim-3 levels and increased CD28 expression on CD8^+^ and Vγ9Vδ2^+^ T cells compared with a single dose of docetaxel treatment in patients with NSCLC (figure 1E-P and online supplemental figure S6A–D). Collectively, the aforementioned findings clearly revealed the therapeutic potential of 1α,25(OH)_2_D_3_ in overcoming cytotoxic T-cell exhaustion and indicated that the decreased vitamin D level in patients with cancer with NSCLC was the etiology of cytotoxic T-cell exhaustion.

10.1136/jitc-2021-003477.supp2 Supplementary data

10.1136/jitc-2021-003477.supp3 Supplementary data

10.1136/jitc-2021-003477.supp4 Supplementary data

10.1136/jitc-2021-003477.supp5 Supplementary data

10.1136/jitc-2021-003477.supp6 Supplementary data

10.1136/jitc-2021-003477.supp7 Supplementary data

10.1136/jitc-2021-003477.supp8 Supplementary data

### Cytokine secretion of T cells in patients with NSCLC treated with rocaltrol was promoted

**Figure 2:**
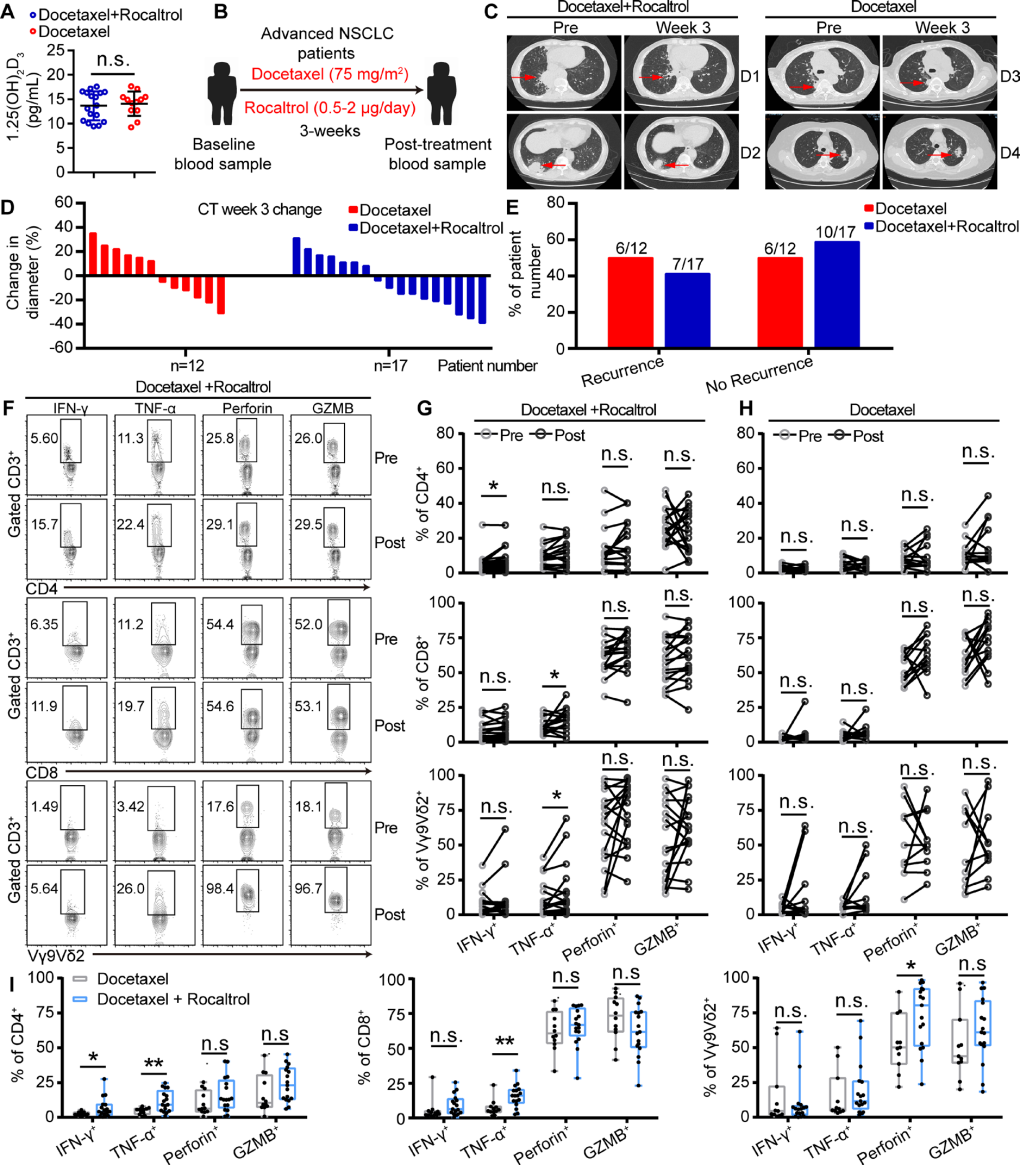
Cytokine secretion of T cells in patients with NSCLC was promoted after rocaltrol treatment. (A) 1α,25(OH)_2_D_3_ levels (baseline) in sera of patients with NSCLC, before therapy with docetaxel and rocaltrol (n=17) or docetaxel (n=12) alone. (B) Overview of study design. Patients with NSCLC received one course of intravenous docetaxel (75 mg/m^2^) followed by treatment with rocaltrol at a dose of 0.5–2.0 µg/day for total of 3 weeks. (C–E) CT scans and waterfall plot showing changes of tumor size in patients with NSCLC who received one course of intravenous docetaxel (75 mg/m^2^), followed by oral rocaltrol at a dose of 0.5–2.0 µg/day for total of 3 weeks. Waterfall plot of percentage of patients with recurrence and no recurrence after treatment with docetaxel and rocaltrol or docetaxel alone. (F–H) Graphs showed the percentage of IFN-γ^+^, TNF-α^+^, perforin^+^, and GZMB^+^ cells in CD4^+^, CD8^+^, and Vγ9Vδ2^+^ T cells of patients with NSCLC, before (pre) and after (post) therapy with docetaxel (75 mg/m^2^) and rocaltrol (0.5–2.0 µg/day) or docetaxel alone at the indicated time points followed by activation of anti-CD3 and anti-CD28 antibodies for 4 hours. Flow cytometry and statistical analysis for the percentage of IFN-γ^+^, TNF-α^+^, perforin^+^, and GZMB^+^ on T cells (CD4^+^, CD8^+^, and Vγ9Vδ2^+^). (I) Graphs showed the proportion of IFN-γ^+^, TNF-α^+^, perforin^+^, and GZMB^+^ in CD4^+^, CD8^+^, and Vγ9Vδ2^+^ T cells from patients with NSCLC, after (post) therapy with docetaxel (75 mg/m^2^) and rocaltrol (0.5–2.0 µg/day) or treatment with docetaxel alone for 3 weeks. n=17 NSCLC donors. (G, H) Paired Student’s t-test. (A, I) Unpaired Student’s t-test. Significance was set to p<0.05 and represented as *p<0.05, **p<0.01, ***p<0.001, ****p<0.0001. GZMB, granzyme B; IFN-γ, interferon gamma; n.s., not significant; NSCLC, non-small cell lung cancer; TNF-α, tumor necrosis factor alpha.

To further explore the role of 1α,25(OH)_2_D_3_ in patients with NSCLC, we analyzed samples from patients with advanced lung cancer receiving rocaltrol (1α,25(OH)_2_D_3_) therapies. In clinical trials of oral rocaltrol, patients with high levels of 1α,25(OH)_2_D_3_ may not benefit from our therapy project, so we excluded them from the clinical trial. Baseline levels of 1α,25(OH)_2_D_3_ in enrolled patients treated with docetaxel and rocaltrol or docetaxel alone were less than 20 pg/mL. The difference of baseline levels of 1α,25(OH)_2_D_3_ between groups of docetaxel or docetaxel combined with rocaltrol was not statistically significant (figure 2A). Patients with NSCLC had received docetaxel or docetaxel combined with rocaltrol treatment at the indicated time points (figure 2B and online supplemental figure S7A). A summary of patients’ information is described in online supplemental table 2 (for cytokine detection). CT scans were performed at the baseline and after treatment with docetaxel and rocaltrol. Changes of tumor size based on the CT imaging after 3 weeks were recorded. Obvious difference of tumor size shrinkage was observed under the treatment of docetaxel combined with rocaltrol (figure 2C-E). Next, we analyzed cytokine secretion of peripheral blood CD4^+^, CD8^+^, and Vγ9Vδ2^+^ T cells during docetaxel or docetaxel combined with rocaltrol therapy in patients with NSCLC. Administration with docetaxel and rocaltrol increased the percentage of IFN-γ^+^CD4^+^, TNF-α^+^CD8^+^, and TNF-α^+^Vγ9Vδ2^+^ T cells and the level of TNF-α in serum compared with that of administration with a single dose of docetaxel (figure 2F–I and online supplemental figure S7B–D) in patients with NSCLC. To assess the percentage of no recurrence patients responding to docetaxel and rocaltrol therapy, we focused our analysis on patients who had at least a 1.2-fold increase in the frequency of IFN-γ^+^ and TNF-α^+^ CD3 T cells. In accordance with our predictions, most of the patients with NSCLC with IFN-γ^+^ and TNF-α^+^ CD3 T cells activated by docetaxel and rocaltrol did not relapse; 1α,25(OH)_2_D_3_ level was marginally increased, but 1α,25(OH)_2_D_3_ level negatively correlated with PD-1 expression on CD8^+^ T cells (online supplemental figures S7E–G). These data suggested that 1α,25(OH)_2_D_3_ treatment might also be important for activation of T cells in patients with cancer with NSCLC.

10.1136/jitc-2021-003477.supp9 Supplementary data

### VDR is a transcription factor for *Pdcd1*, *Tim3* and *Tigit* genes

**Figure 3:**
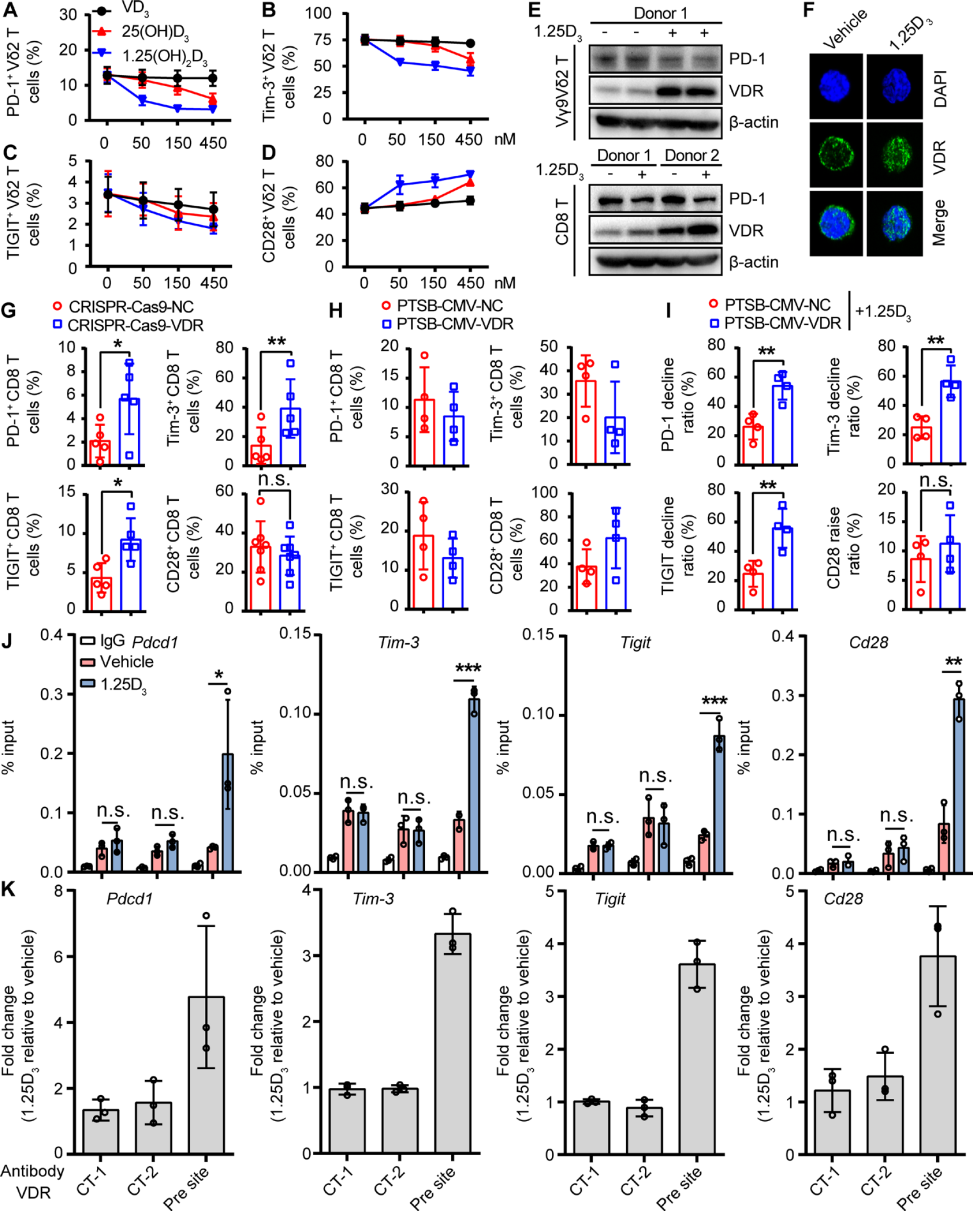
VDR is a transcription factor for *PD-1*, *Tim3* and *Tigit* genes. (A–D) Vγ9Vδ2^+^ T cells were treated with vitamin D (VD3, 25(OH)D_3_, 1α,25(OH)_2_D_3_) in fresh medium at the indicated concentration for 48 hours. Percentages of PD-1^+^ (A), Tim-3^+^ (B), TIGIT^+^ (C), and CD28^+^ (D) on Vγ9Vδ2^+^ T cells are shown. n=3 healthy donors. (E) Protein expression of VDR and PD-1 in vehicle-treated or 1α,25(OH)_2_D_3_ (50 nM)-treated human healthy CD8^+^ and Vγ9Vδ2^+^ T cells for 48 hours. (F) Confocal imaging of VDR (green) and nucleus (blue) in Vγ9Vδ2^+^ T cells after treatment with vehicle or 1α,25(OH)_2_D_3_ (50 nM) for 12 hours. (G) Cell-surface expression of PD-1^+^, Tim-3^+^, TIGIT^+^, and CD28^+^ on CD8^+^ T cells which were selected from the CD8^+^ T cells of *VDR* knockout (CRISPR-Cas9-*VDR*) or control vector (CRISPR-Cas9-NC). n=5–7 healthy donors. (H, I) Effects of *VDR* overexpression on level of PD-1^+^, Tim-3^+^, TIGIT^+^, and CD28^+^ on activated CD8^+^ T cells. Human CD8^+^ T cells were transduced by PTSB–CMV–GFP–*VDR* or vector control (PTSB–CMV–GFP–NC) using a lentivirus followed by 1α,25(OH)_2_D_3_ (50 nM) treatment two times at 1-day intervals. Statistical analysis for the decline ratio of PD-1^+^, Tim-3^+^, and TIGIT^+^ and raise ratio of CD28^+^ levels on CD8^+^ T cells are shown. n=4 healthy donors. (J, K) Graph shows ChIP-quantitative real-time PCR analysis of VDR at the promoter of *PD-1*, *Tim-3*, *Tigit*, and *Cd28*. The relative enrichment of VDR binding to the promoter of *PD-1*, *Tim-3*, *Tigit*, and *Cd28* in vehicle or 1α,25(OH)_2_D_3_ (50 nM) pretreated CD8^+^ T cells for 12 hours is shown (pre site=predicted binding site; CT-1, CT-2=non-binding sites as control). VDR levels were normalized to the input. n=3 healthy donors. Data represent mean±SD, unpaired Student’s t-test (G, I, J). Significance was set to p<0.05 and represented as *p<0.05, **p<0.01, ***p<0.001, and ****p<0.0001, n.s., not significant; PD-1, programmed cell death-1; TIGIT, T-cell immunoreceptor with Ig and ITIM domains; Tim-3, T-cell immunoglobulin and mucin-domain containing-3; VDR, vitamin D receptor.

The canonical action pathway of vitamin 1α,25(OH)_2_D_3_ is through binding to its receptor (VDR), which translocates into nucleus and functions as a transcription factor.^21^ We used distinct active forms of vitamin VD_3_, 25(OH)D_3_, and 1α,25(OH)_2_D_3_ (low, moderate, and high activities) to stimulate T cells, respectively. The results showed that ICR levels were declined maximum by 1α,25(OH)_2_D_3_, which indicated that the expression of ICRs on T cells was regulated mainly by 1α,25(OH)_2_D_3_ (figure 3A–D and online supplemental figures S8A, B). Indeed, 1α,25(OH)_2_D_3_ treatment increased VDR expression and promoted its nuclear translocation in cytotoxic T cells (figure 3E, F). Thus, we next investigated whether VDR was involved in regulating the expression of ICRs. VDR-KO CD8^+^ T cells were constructed using CRISPR-Cas9 targeted approach and the results demonstrated that knockout of VDR remarkably potentiated the expression of PD-1, TIGIT, and Tim-3, whereas CD28 level was marginally reduced (figure 3G and online supplemental figure S9A–C). Consistently, VDR overexpression in CD8^+^ T cells were constructed using PTSB–CMV–VDR approach, and the results showed that the expression of PD-1, TIGIT, and Tim-3 were inhibited under stronger 1α,25(OH)_2_D_3_/VDR signaling, whereas CD28 expression was either unchanged or only marginally increased (figure 3H, I, and online supplemental figure S9D). Thus, we hypothesized that VDR might be a transcription factor that inhibits the transcription of *Pdcd1*, *Tigit*, and *Tim3* genes. Besides, we used the JASPAR website to predict VDR binding sites in the promoter region of *Pdcd1*, *Tigit*, *Tim3*, and *Cd28* genes (online supplemental figure S9E), and ChIP-qPCR analysis showed that 1α,25(OH)_2_D_3_ administration notably promoted the binding of VDR to these three genes (figure 3J, K). Collectively, the aforementioned results demonstrated that VDR is an inhibitory transcription factor for *Pdcd1*, *Tigit*, and *Tim3* in cytotoxic T cells.

10.1136/jitc-2021-003477.supp10 Supplementary data

10.1136/jitc-2021-003477.supp11 Supplementary data

### 1α,25(OH)_2_D_3_ promotes H3K27 acetylation in the promoter of *Cd28* and DNA methylation in CpG islands of *Pdcd1* gene

**Figure 4:**
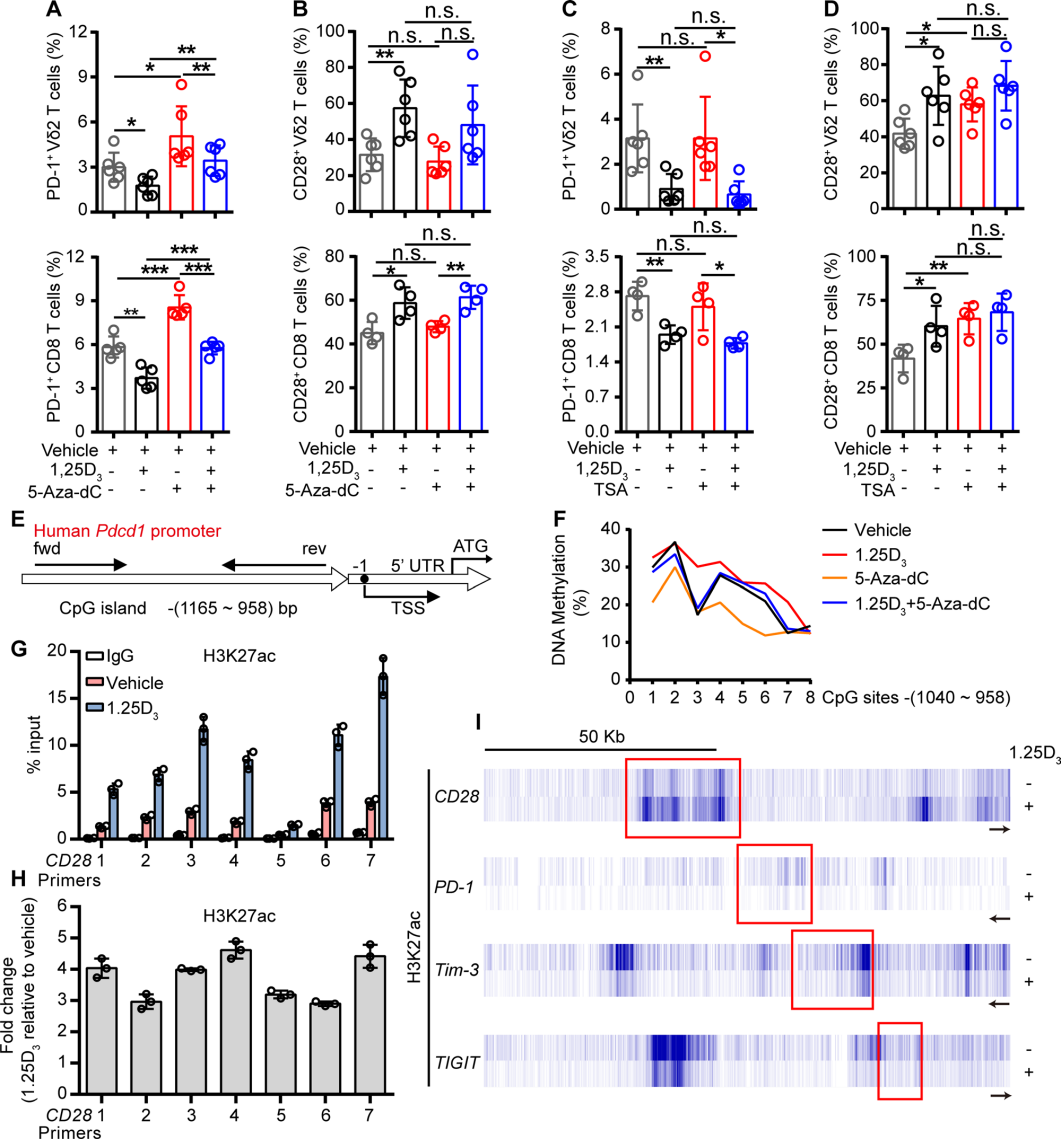
1α,25(OH)_2_D_3_ promotes H3K27 acetylation in the promoter of *Cd28* and DNA methylation in CpG islands of *Pdcd1* promoter region. (A, B) Percentage of PD-1^+^ and CD28^+^ T cells (Vγ9Vδ2, CD8 T) after treatment with 5′-aza-2′-deoxycytidine (5-aza-dC, 1 µM), 1α,25(OH)_2_D_3_, or the combination. (C, D) Frequency of PD-1^+^ and CD28^+^ T cells (Vγ9Vδ2, CD8 T) after treatment with TSA (100 nM), 1α,25(OH)_2_D_3_ or the combination. Cells were treated with 5-aza-dC, TSA, 1α,25(OH)_2_D_3_ (50 nM), or the combination for 48 hours. (Vγ9Vδ2 T, n=6; CD8 T, n=4 healthy donors). (E) Graph showing the position of CpG island in the *Pdcd-1* promoter. (F) Vγ9Vδ2^+^ T cells were treated with 1α,25(OH)_2_D_3_ (50 nM), 5-Aza-dC (1 µM), 1α,25(OH)_2_D_3_+5-Aza-dC, or vehicle. The frequency of DNA methylation at the CpG islands in *Pdcd-1* promoter regions was detected by pyrosequencing. (G, H) Graph showing ChIP-seq analysis of histone 3 lysine 27 acetylation (H3K27ac) at *Cd28* promoter. H3K27ac levels were normalized to the input. n=3 healthy donors. Primers 1–7 (quantitative real-time PCR primers for CD28, S1–S7). (I) ChIP-seq for histone acetylation (H3K27ac) of indicated gene in Vγ9Vδ2^+^ T cells with 1α,25(OH)_2_D_3_ treatment or not. Vγ9Vδ2^+^ T cells from healthy donors were stimulated with 1α,25(OH)_2_D_3_ (50 nM) two times at 1-day intervals. Data represent mean±SD. Unpaired Student’s t-test (A–D). Significance was set to p<0.05 and represented as *p<0.05, **p<0.01, ***p<0.001, and ****p<0.0001. n.s., not significant; PD-1, programmed cell death-1; TSA, trichostatin A; TSS, transcription start site.

To further investigate how the 1α,25(OH)_2_D_3_/VDR pathway regulate the expression of ICRs and costimulatory molecule, we tried to find out the coactivator or corepressor proteins that may interact with VDR in Vγ9Vδ2^+^ T cells, using coimmunoprecipitation and mass spectrometer methods. The results showed that VDR could interact with multiple different types of histones. Previous studies have shown that VDR also regulates gene expression through epigenetic modifications.^31 32^ So, we next checked whether the regulation of ICRs by 1α,25(OH)_2_D_3_ was dependent on epigenetic modification. In order to do so, Vγ9Vδ2^+^ T cells or CD8^+^ T cells were cultured in the presence or absence of DNA methyltransferase inhibitor decitabine (5-Aza-dC) or histone deacetylase inhibitor trichostatin A (TSA) during 1α,25(OH)_2_D_3_ treatment. Interestingly, the inhibition of PD-1 in response to 1α,25(OH)_2_D_3_ was reversed by 5-Aza-dC but not TSA, while CD28 was exact opposite: its expression was induced by TSA but not 5-Aza-dC (figure 4A–D and online supplemental figure S10A–D). The inhibition of Tim-3 and TIGIT by 1α,25(OH)_2_D_3_, on the other hand, was largely unaffected by either 5-Aza-dC or TSA (online supplemental figure S10E, F). These results suggested that the inhibition of PD-1 by 1α,25(OH)_2_D_3_ was dependent on DNA methylation, and promotion of CD28 was dependent on histone acetylation. Indeed, 1α,25(OH)_2_D_3_ treatment promoted the methylation of CpG sites of the *Pdcd1* gene (figure 4E, F). Moreover, ChIP-qPCR analysis further confirmed the upregulation of H3K27 acetylation in the *Cd28* gene but not in *Pdcd1*, *Tim3*, or *Tigit* (figure 4G-I). Taken together, these results corroborated that 1α,25(OH)_2_D_3_/VDR signaling adopted epigenetic approaches to decrease the expression of inhibitory checkpoint receptors and to promote costimulatory CD28 expression.

10.1136/jitc-2021-003477.supp12 Supplementary data

### VDR mediates Ca^2+^ influx to promote cytokine production of activated T cells

**Figure 5:**
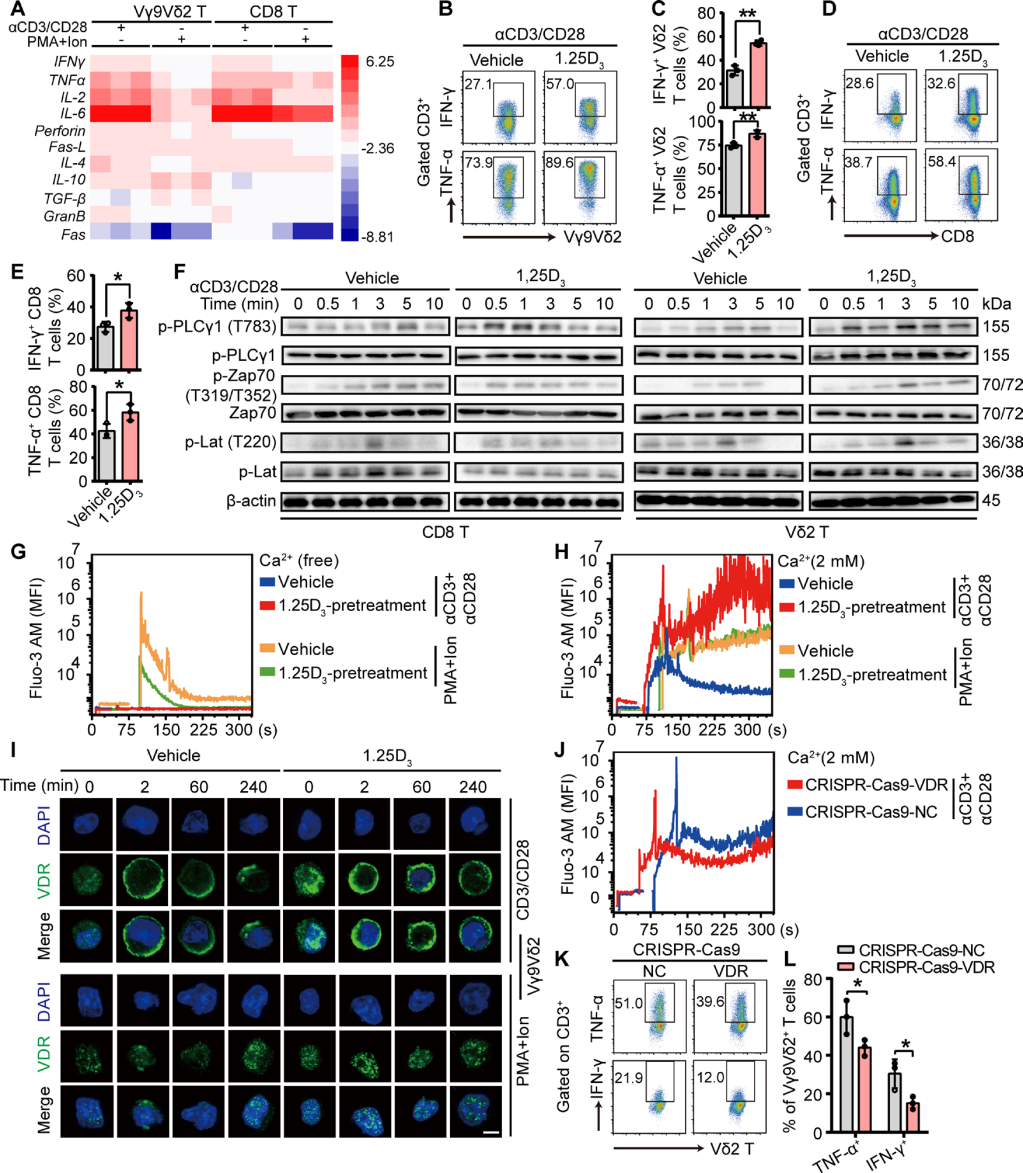
VDR promotes calcium influx-mediated cytokine production under TCR activation. (A) Human Vγ9Vδ2^+^ and CD8^+^ T cells were pretreated with 1α,25(OH)_2_D_3_ (50 nM) or vehicle, followed by anti-human CD3/CD28 antibody stimulation for 4 hours. qPCR for the mRNA expression of cytokines and their fold changes are shown in hot map. Experimental procedures are described in the Materials and Methods section. (B–E) Human Vγ9Vδ2^+^ (B, C) and CD8^+^ (D, E) T cells were treated with vehicle or 1α,25(OH)_2_D_3_ (50 nM) two times at 2-day intervals, followed by restimulation with anti-human CD3/CD28 antibodies for another 4 hours. Flow cytometry and statistical analysis were performed for the percentage of T cells producing TNF-α and IFN-γ. n=3 healthy donors. (F) Immunoblotting analysis of protein phosphorylation in 1α,25(OH)_2_D_3_ pretreated T cells (CD8^+^, Vγ9Vδ2^+^) in response to treatment with TCR activation. (G, H) Vγ9Vδ2^+^ T cells were preloaded with fluo-3 AM and then supplemented with Ca^2+^ or Ca^2+^ free medium. Cells were then treated with anti-human CD3/CD28 or PMA and Ion for indicated time. Ca^2+^ fluorescence intensity transformation was detected by flow cytometry at the indicated time points. Human Vγ9Vδ2^+^ T cells were treated with vehicle or 1α,25(OH)_2_D_3_ (50 nM) two times at 2-day intervals (I) Confocal images of VDR (green) expression and distribution. Vehicle or 1α,25(OH)_2_D_3_ pretreated Vγ9Vδ2 T cells were stimulated with anti-human CD3/CD28 antibodies or PMA plus Ionomycin for 0, 2, 60, and 240 min, and images were captured with a laser scanning confocal microscope. Scale bars: 5 µm. (J-L) VDR knockout Vγ9Vδ2^+^ T cells were generated by CRISPR-Cas9 technology, and then stimulated with anti-human CD3/CD28 antibodies at the indicated time points. Ca^2+^ ions fluorescence intensity transformation (J), and the percentage of Vγ9Vδ2^+^ T cells producing TNF-α and IFN-γ (K, L) were detected by flow cytometry. Data represent mean±SD. Unpaired Student’s t-test (C, E, L). *P<0.05, **P<0.01, ***P<0.001, ****P<0.0001. IFN-γ, interferon gamma; IL, interleukin; n.s., not significant; PMA, phorbol 12-myristate 13-acetate; TCR, T-cell receptor; TNF-α, tumor necrosis factor alpha; VDR, vitamin D receptor; MFI, mean fluorescence intensity.

From the aforementioned data, we demonstrated that 1α,25(OH)_2_D_3_ could downregulate expression of inhibitory checkpoint receptors on T cells, and release T-cell inhibition, so we wondered if 1α,25(OH)_2_D_3_ promote their cytokine production. To investigate whether 1α,25(OH)_2_D_3_ promoted cytokine production of T cells in response to TCR stimulation in vitro, we cultured Vγ9Vδ2^+^ T lymphocytes and CD8^+^ T cells from PBMCs of healthy donors in the presence or absence of 1α,25(OH)_2_D_3_ treatment. The expression of cytokine-related genes (figure 5A) in Vγ9Vδ2^+^ and CD8^+^ T cells was detected with quantitative real-time PCR, and the intracellular level of cytokines was determined by flow cytometry. The results showed that 1α,25(OH)_2_D_3_ treatment enhanced Th1 cytokine production of Vγ9Vδ2^+^ (figure 5B, C) and CD8^+^ (figure 5D, E) T cells in response to TCR activation (figure 5F and online supplemental figure S11A) and also promoted CD107a degranulation (online supplemental figure S11B) but had no obvious effects on expression of Fas, perforin, and NKG2D (online supplemental figures S11C–E). Interestingly, production of IFN-γ and TNF-α in response to 1α,25(OH)_2_D_3_ treatment was insignificantly altered under PMA and ionomycin stimulation (online supplemental figures S12A, B), which suggested that the enhanced Th1 cytokine production in 1α,25(OH)_2_D_3_ pretreated T cells was associated with TCR recognition. To determine whether Ca^2+^ ions were involved in regulating IFN-γ and TNF-α production in 1α,25(OH)_2_D_3_ pretreated Vγ9Vδ2 T cells, the intracellular Ca^2+^ ions were chelated with BAPTA-AM, and the results showed a significant decrease in the percentage of IFN-γ and TNF-α production (online supplemental figure S13). Besides, we found that stimulation of anti-CD3 and anti-CD28 antibodies was incapable of inducing intracellular Ca^2+^ ions release in activated T cells (figure 5G). However, supplementation of Vγ9Vδ2^+^ T cells with 2 mM Ca^2+^ ions resulted in sustainable Ca^2+^ ions influx in 1α,25(OH)_2_D_3_ pretreated T cells under the brief stimulation of the TCR (figure 5H). Importantly, our results found that stimulation with TCR in vitro promoted VDR migration from cytoplasm and nucleus to the membrane of T cells (figure 5I). Therefore, we hypothesized that VDR might need to migrate to the cell surface in order to efficiently mediate Ca^2+^ ion influx in cytotoxic T cells. We next checked whether VDR was involved in regulating Ca^2+^ ion influx-mediated cytokine production in cytotoxic T cells. Hence, we adopted CRISPR-Cas9 technology to knockout VDR in Vγ9Vδ2^+^ T cells and found that both of VDR and Ca^2+^ ions (figure 5J) were necessary for production of IFN-γ and TNF-α (figure 5K, L), and Ca^2+^ ions influx is VDR dependent. These data demonstrated that VDR mediates Ca^2+^ ion influx to enhance cytokine production in activated T cells.

10.1136/jitc-2021-003477.supp13 Supplementary data

10.1136/jitc-2021-003477.supp14 Supplementary data

10.1136/jitc-2021-003477.supp15 Supplementary data

### 1α,25(OH)_2_D_3_ promotes antitumor immunity of cytotoxic T cells

**Figure 6:**
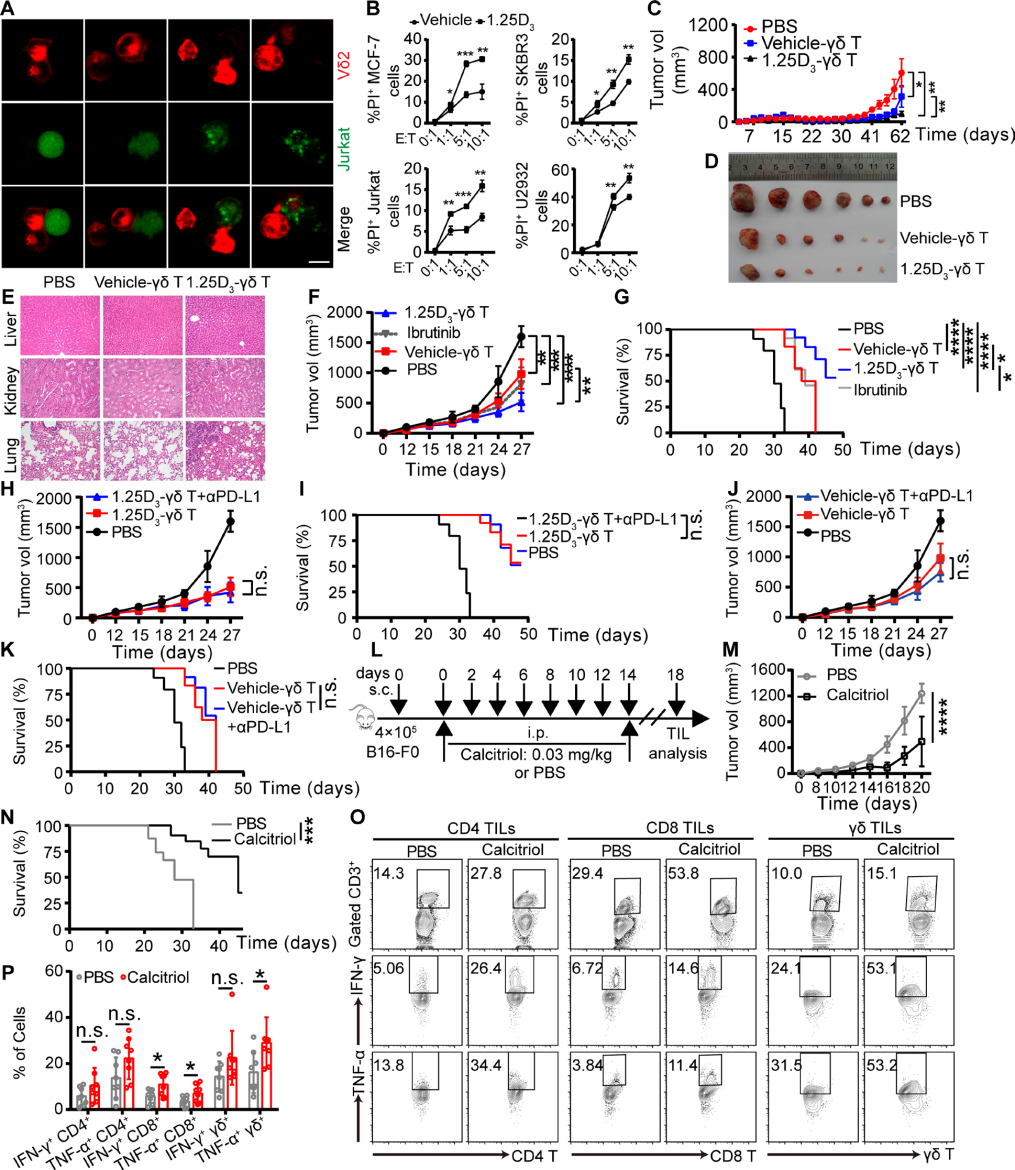
1α,25(OH)_2_D_3_ promotes antitumor immunity of cytotoxic T cells. (A, B) Vγ9Vδ2 T cells (effector, red) pretreated with 1α,25(OH)_2_D_3_ were incubated with MCF-7, SKBR3, or Jurkat cells (target, green) at different E:T ratios for 6 hours; the confocal images were captured (A); and the percentages of dead cells out of total target cells identified as PI^+^ are shown (B), n=3 healthy donors. Scale bars, 20 µm. (C) MCF-7 model. Graphs showing tumor growth, represented by TV, n=6 mice per group. (D) Images of MCF-7 tumors in mice at day 65 post-tumor implantation, n=10 mice per group. (E) Tissue pathology of the indicated organs was evaluated by H&E staining at the end of the experiment (days 31 after Vγ9Vδ2 T cell infusion). Original magnification: ×100. (F–K) U2932 model. TV (F, H, J) and survival curves from data in (G, I, K). n=6 mice per group. (L) Experimental approach: subcutaneous and intraperitoneal. (M, N) Progression of B16-F0 melanomas in mice and the survival of mice were assessed in wild-type mice. n=8 mice. (O, P) Tumor-infiltrating T cells were isolated and analyzed on day 18. Calcitriol (1α,25(OH)_2_D_3_). The level of cytokines secreted by tumor-infiltrating CD4^+^, CD8^+^, and γδ^+^ T cell subsets was measured on day 18. n=8 mice. Experiments were independently repeated three times (C–K). Data represent mean±SD. Unpaired Student’s t-test (B); two-way analysis of variance (C, F, H, J, M); log-rank (Mantel-Cox) test was used in (G, I, K, N). *P<0.05, **P<0.01, ***P<0.001, ****P<0.0001. E:T, effector:target; IFN-γ, interferon gamma; IL, interleukin; n.s., not significant; TCR, T-cell receptor; TIL, tumor infiltrating lymphocyte; TNF-α, tumor necrosis factor alpha; TV, tumor volume; VDR, vitamin D receptor.

Recent clinical studies also underscore the safety and efficacy of vitamin C mediated allogeneic Vγ9Vδ2 T-cell immunotherapy in patient with late-stage lung or liver cancer.^10^ Since 1α,25(OH)_2_D_3_ could reverse the exhaustion phenotype of cytotoxic T cells, we assumed that it might also regulate antitumor immunity mediated by cytotoxic T cells. To test this assumption, 1α,25(OH)_2_D_3_ treated Vγ9Vδ2^+^ T cells were cocultured with multiple lines of CFSE-labeled tumor cells for 6 hours and the apoptosis of tumor cells were analyzed. Vγ9Vδ2^+^ T cells recognize and lead to tumor cell lysis directly (figure 6A). As expected, 1α,25(OH)_2_D_3_ treated Vγ9Vδ2^+^ T cells showed increased cytotoxicity (figure 6B and online supplemental figure S14A, B). To examine whether 1α,25(OH)_2_D_3_ treatment would also be necessary for reinvigoration of antitumor responses of Vγ9Vδ2^+^ T cells, NOD-SCID mice were inoculated with MCF-7 (online supplemental figure S15A) and U2932 cells (online supplemental figure S15B). We tracked tumor growth by measuring TV and observed that 1α,25(OH)_2_D_3_ pretreated Vγ9Vδ2^+^ T cells could slow tumor growth in MCF-7 tumor model (figure 6C, D). Besides, it should be noted that antitumor immunity mediated by 1α,25(OH)_2_D_3_ pretreated Vγ9Vδ2^+^ T cells had no side effects on organs, including the liver, kidneys, and lungs, displaying the safety of Vγ9Vδ2 T cells (figure 6E). We also found that 1α,25(OH)_2_D_3_ pretreated Vγ9Vδ2^+^ T cells showed enhanced antitumor immunity in adoptive therapy, and they even provided better protection than ibrutinib in treating diffuse large B-cell lymphoma (DLBCL) U2932 (figure 6F, G). However, in tumor-bearing mice models, no obvious difference of tumor size shrinkage was observed under the therapy of 1α,25(OH)_2_D_3_ pretreated Vγ9Vδ2^+^ T cells combined with αPD-L1 (figure 6H-K and online supplemental figure S15C, D). To further investigate the physiological function of 1α,25(OH)_2_D_3_ (calcitriol), we next used mouse B16-F0 melanoma models to investigate the effect of calcitriol in antitumor immunity. The results showed that mice treated with calcitriol had slower tumor progression than PBS treated mice (figure 6L–N). Moreover, administration with calcitriol led to more production of IFN-γ and TNF-α in tumor-infiltrating CD8^+^ and γδ^+^ T cells (figure 6O, P). Analysis of CD8^+^ and γδ^+^ T cells isolated from tumor tissues showed that the expression of PD-1 was obviously reduced in mice treated with calcitriol (online supplemental figure S15E). These results indicated that 1α,25(OH)_2_D_3_ treatment reversed exhaustion and enhanced T-cell cytotoxicity both in vitro and in vivo.

10.1136/jitc-2021-003477.supp16 Supplementary data

10.1136/jitc-2021-003477.supp17 Supplementary data

## Discussion

From the immunological point of view, tumor growth and progression are due to imperfect immune surveillance and failure of cancerous cell eradication. Tumor cells evade immune surveillance through different mechanisms, including activation of different immune checkpoint pathways that suppress antitumor immune responses. ICR-targeted therapies such as blockade of PD-1/PD-L1/CTLA4 reinvigorate antitumor immune responses and promote immune-mediated elimination of tumor cells.^33^ Some of these approaches have been approved for certain cancer treatments, and hundreds more are under clinical trials. However, accumulating evidence showed that only a fraction of patients with cancer benefit from ICR-targeted therapies. This is probably because cancer cells adopt multiple immune suppressing approaches and only a fraction of patients show increased expression of a certain ICR. Thus, combined ICR therapies were developed to increase blockade efficacy. Our study demonstrated that 1α,25(OH)_2_D_3_/VDR signaling suppresses the expression of multiple ICRs including PD-1, TIGIT, and Tim-3 in cytotoxic T cells, and enhanced their antitumor activity. 1α,25(OH)_2_D_3_ treatment also reversed the decreased costimulatory CD28 expression on them. Besides, we demonstrated that the mechanism of 1α,25(OH)_2_D_3_/VDR in regulating antitumor immunity of T cells is associated with rescuing their exhausted phenotype and VDR-mediated Ca^2+^ ions influx of human cytotoxic T cells. These results suggested that 1α,25(OH)_2_D_3_/VDR-targeted therapy might have broader scope of application.

Another obstruction that hinders the application of ICR-targeted therapy is severe immune-related adverse events, which arise in some patients with cancer.^34^ These therapies probably cause imbalance of the normal physiological barriers against autoimmunity and lead to various local and systemic autoimmune responses. In contrast, vitamin D possesses immune-suppressing function in inflammatory responses elicited by innate immune cells or CD4 T cells.^23^ Besides, vitamin D also favors the differentiation of Foxp3^+^ Treg cells,^35^ which are potent suppressors in controlling autoimmune responses. These studies suggested that vitamin D treatment might be much safer with low risks of systemic inflammatory storm and autoimmunity. This needs further clinical validation in the future. In addition, vitamin D also directly suppresses the proliferation and induces apoptosis of a variety types of tumors.^27^ Epidemiological surveys have revealed that low level of vitamin D is associated with high risks of tumor, and vitamin D supplementation reduces the risk of cancer death.^30 36 37^ These observations potentiated the rationale for vitamin D therapy in cancer.

Interestingly, some studies showed the reduction of ICRs by 1α,25(OH)_2_D_3_ only occurred on cytotoxic T cells but not on CD4^+^ T cells, suggesting different ways of action of vitamin D in these cell subtypes. The precise mechanisms underlying these different actions need further investigation. Previous studies also revealed the divergent role of vitamin D on PD-1 expression. Bendix *et al* found that long-term administration of vitamin D3 on patients with Crohn’s disease promoted PD-1 expression on CD4^+^ CD25^int^ T cells,^38^ and Sheikh *et al* showed that stimulation of CD4^+^ T cells with vitamin D3 in vitro enhanced the expression of PD-1.^39^ In contrast, Pincikova *et al* reported that vitamin D treatment on patients with cystic fibrosis was associated with reduced PD-1 expression on both CD4^+^ and CD8^+^ T cells.^40^ Our results showed that 1α,25(OH)_2_D_3_ could reverse exhaustion of CD4 T cells derived from PBMCs of healthy donors in vitro. These findings suggest that the physical condition of patients may influence the action of vitamin D, and the effects of vitamin D in patients with different tumors need to be validated.

Vitamin 1α,25(OH)_2_D_3_ binds to VDR, which triggers the nuclear translocation of VDR and regulates the transcription of multiple genes.^21^ In this study, we report that VDR activation by 1α,25(OH)_2_D_3_ induces transcriptional suppression of *Pdcd1*, *Tim3*, and *Tigit* genes in cytotoxic T cells. However, how VDR mediates the inhibition of transcription of these genes is unknown. Previous studies have revealed that blimp-1^41^ and T-bet^42^ are inhibitory transcription factors for PD-1. Does VDR activation promote recruitment of these factors to Pdcd1 gene locus? VDR frequently forms a heterodimer with retinoid X receptor for DNA binding.^43^ Epigenetic modification such as DNA methylations and histone modifications were also involved in PD-1 regulation.^44^ Xu *et al* reported that DNA modification 5-methylcytosine at CpG islands in gene promoters or at transcriptional enhancers was related with silence of gene expression.^45^ Some studies demonstrated that two CpG-rich regions upstream of *Pdcd1* transcription start site (TSS) were dynamically methylated in CD8 T cells during infection of acute lymphocytic choriomeningitis virus (LCMV) or chronic HIV.^46 47^ On a genomic level, promoters were enriched histones such as H3 lysine 27 acetylation (H3K27ac) and were generally considered as promoted gene expression.^48^ When PD-1 expression was enhanced on CD8 T cells in vitro, H3K27ac and H3K9ac were located at CR-C (this element, which are located 100 bp and 1.1 kb upstream of the TSS, contains multiple transcription factor binding sites).^41^ In addition, the CR-C region of *Pdcd1* was enriched with repressive histone modification H4K20me3, H3K27m3, and H3K9me3 to inhibit PD-1 expression.^49^ 1α,25(OH)_2_D_3_ reduced the level of H3K27 acetylation at the promoter of *Pdcd1*, indicating that histone modification might be also involved in regulation of PD-1 expression. Our results also suggested that the inhibition of PD-1 expression by 1α,25(OH)_2_D_3_ was dependent on DNA methylation, but the function relevance for DNA methyltransferase is unclear yet, and how methylation and acetylation coordinately regulate the expression of ICRs and costimulatory molecules deserved further study. VDR also recruits co-regulatory complexes for chromatin remodeling, histone modification and RNA polymerase II activation.^50^ Whether these elements coordinate to mediate transcriptional suppression of *Pdcd1*, *Tim3*, and *Tigit* genes needs further investigation. Therefore, it is important to recognize that 1α,25(OH)_2_D_3_ molecule has broad pleiotropic effects, and other pathways are probably implicated.

T cells are central players in immune responses against exogenous pathogens and endogenous cancers. T-cell activation requires two signals: major antigen-induced signal from the TCR and a secondary signal from costimulatory receptors.^51^ A previous study has shown that the TCR, Ca^2+^, and CD28 together form a dual-positive-feedback circuit that substantially amplifies T-cell signaling and thus increases antigen sensitivity.^52^ Using VDR-knockout Vγ9Vδ2^+^ T cells, we demonstrated that VDR deficiency in T cells resulted in decreased extracellular Ca^2+^ ions influx and reduced IFN-γ and TNF-α production in the context of αCD3/CD28 stimulation. Our studies found that 1α,25(OH)_2_D_3_ regulated the cytokine production of T cells through VDR and Ca^2+^ influx. Interestingly, we discovered that VDR in T cells could migrate from cytoplasm and nucleus to cell membrane in response to αCD3/CD28 treatment. However, the mechanisms of how VDR migrated to the cell membrane and how they worked there remain to be investigated.

Currently, most immune cell therapy strategies, such as CAR-T, are mainly based on αβ T cells (CD4 and CD8 T) and have achieved ground-breaking success in CD19^+^ diseases,^11 53^ but it is associated with unique acute toxicities, which can be severe or even fatal for patients, the application of such a strategy have shown limited success in treating solid tumors.^54^ Notably, the published article has intensively reviewed the clinical potential of allogeneic Vγ9Vδ2 T cells as a new strategy for cancer immunotherapy, showing the safety of Vγ9Vδ2 T cells.^10 55^ Previous reports have proven that T-cell exhaustion is a poor responsive status with an upregulated level of ICRs, decreased production of antitumor-related Th1 cytokines, and suppressed antitumor efficacy in patients with cancer.^33 56^ Interestingly, recent studies also show that tumor mutation burden, microsatellite instability, and immune microenvironment (PD-1 and PD-L1 expressions) are prognostic factors associated with better patient survival in ICR inhibitor-targeted therapies.^56–60^ Vitamin 1α,25(OH)_2_D_3_ downregulated ICRs, increased expression of antitumor-related cytokines by activated T cells, and elevated antitumor activity in vitro and in vivo. We found that the proportion of no recurrence patients treated with the combination of docetaxel and rocaltrol was slightly higher than that of patients treated with docetaxel alone. Considering the number of enrolled patients and the lack of observation time for treatment effects, we expect more patients to be enrolled in clinical trials and observed for a longer time to fully evaluate the effect of this therapy. Our study therefore expands on the knowledge of 1α,25(OH)_2_D_3_ biology and highlights the clinical potential of the regulation of antitumor immunity through the 1α,25(OH)_2_D_3_/VDR pathway.

In summary, our study demonstrated that 1α,25(OH)_2_D_3_ reduced expression of multiple ICRs and increased expression of costimulatory molecule CD28 through modification of their gene promoter regions, enhanced production of Th1 cytokines through VDR-mediated Ca^2+^ ion influx signaling pathway, and facilitated antitumor immunity by cytotoxic T cells. Primary clinical evidence also supported our findings. Taken together, 1α,25(OH)_2_D_3_ and VDR-targeted therapy might be a safe and economical approach with pleiotropic effects and broad application scope to promote antitumor responses of patients with cancer. 1α,25(OH)_2_D_3_ combined with some ICR inhibitors or drugs may have better efficacy.

10.1136/jitc-2021-003477.supp18 Supplementary data

## Data Availability

Data are available in a public, open access repository.
