# Supplementary material for: 1α,25(OH)2D3 reverses exhaustion and enhances antitumor immunity of human cytotoxic T cells
Source: J Immunother Cancer. 2022 Mar 22;10(3):e003477. doi: 10.1136/jitc-2021-003477 (PMC8943781; doi:10.1136/jitc-2021-003477)
Supplement: Supplementary data [file jitc-2021-003477supp001.pdf]

Supplementary Tables:

Table 1

Patient characteristics										Healthy donor characteristics		
Case ID	Gender	Age	Types of cancer	Stage at diagnosis	Case ID	Gender	Age	Types of cancer	Stage at diagnosis	Case ID	Gender	Age
1	female	63	NSCLC	IV	48	male	84	NSCLC	IV	1	male	55
2	female	63	NSCLC	IV	49	male	60	NSCLC	IV	2	male	49
3	male	46	NSCLC	IV	50	male	51	NSCLC	IV	3	male	53
4	male	55	NSCLC	IV	51	male	62	NSCLC	IV	4	male	72
5	female	57	NSCLC	IV	52	female	65	NSCLC	IV	5	female	49
6	male	65	NSCLC	IV	53	female	38	NSCLC	IV	6	female	60
7	female	50	NSCLC	IV	54	female	82	NSCLC	IV	7	female	62
8	male	49	NSCLC	IV	55	male	58	NSCLC	IV	8	male	64
9	male	68	NSCLC	IV	56	female	60	NSCLC	IV	9	male	45
10	female	37	NSCLC	III	57	male	49	NSCLC	IV	10	male	33
11	female	58	NSCLC	III	58	female	63	NSCLC	IV	11	male	53
12	female	78	NSCLC	IV	59	male	42	NSCLC	IV	12	female	57
13	male	28	NSCLC	IV	60	female	79	NSCLC	IV	13	male	55
14	female	36	NSCLC	IV						14	male	66
15	male	58	NSCLC	IV						15	male	68
16	female	62	NSCLC	IV						16	female	54
17	female	70	NSCLC	IV						17	male	59
18	female	67	NSCLC	IV						18	male	61
19	female	53	NSCLC	IV						19	male	64
20	female	56	NSCLC	IV						20	female	28
21	female	45	NSCLC	IV						21	male	51
22	female	56	NSCLC	IV						22	female	44
23	male	39	NSCLC	IV						23	male	47
24	male	63	NSCLC	IV						24	female	45
25	female	46	NSCLC	IV						25	female	66
26	male	60	NSCLC	IV						26	male	58
27	female	64	NSCLC	IV						27	male	57
28	male	68	NSCLC	IV						28	male	50
29	male	60	NSCLC	IV						29	female	55
30	male	69	NSCLC	IV						30	male	49
31	male	15	NSCLC	IV						31	male	53
32	female	55	NSCLC	IV						32	male	34
33	female	51	NSCLC	IV						33	female	39
34	female	44	NSCLC	IV						34	female	41
35	female	64	NSCLC	IV						35	male	46
36	female	52	NSCLC	IV						36	female	50
37	male	55	NSCLC	IV						37	male	60
38	male	62	NSCLC	IV						38	male	63
39	male	58	NSCLC	IV						39	male	64
40	female	78	NSCLC	IV						40	male	71
41	male	54	NSCLC	IV						41	male	59
42	female	81	NSCLC	IV						42	male	58
43	male	59	NSCLC	IV						43	male	60
44	female	70	NSCLC	IV						44	male	33
45	male	29	NSCLC	IV						45	female	46
46	male	55	NSCLC	IV						46	male	49
47	male	40	NSCLC	IV						47	male	56

Table 2

For cell-surface markers detection											
Treatment group						Randomized controlled trial					
Case ID	Gender	Age	Types of cancer	Stage at diagnosis	Docetaxel +Rocaltrol (day)	Case ID	Gender	Age	Types of cancer	Stage at diagnosis	Docetaxel without Rocaltrol (day)
1	female	59	NSCLC	IV	21	1	female	39	NSCLC	IV	21
2	male	50	NSCLC	IV	21	2	female	83	NSCLC	III	21
3	male	53	NSCLC	IV	21	3	female	61	NSCLC	IV	21
4	female	73	NSCLC	III	21	4	male	64	NSCLC	IV	21
5	male	61	NSCLC	III	21	5	male	53	NSCLC	IV	21
6	female	52	NSCLC	III	21	6	female	76	NSCLC	IV	21
7	male	45	NSCLC	IV	21	7	female	62	NSCLC	IV	21
8	male	60	NSCLC	IV	21	8	female	40	NSCLC	III	21
9	male	66	NSCLC	IV	21	9	male	65	NSCLC	IV	21
10	male	45	NSCLC	IV	21	10	male	53	NSCLC	III	21
11	male	80	NSCLC	IV	21	11	male	58	NSCLC	IV	21
12	female	61	NSCLC	IV	21	12	male	65	NSCLC	IV	21
13	male	61	NSCLC	IV	21	13	female	61	NSCLC	IV	21
14	female	54	NSCLC	III	21	14	female	34	NSCLC	IV	21
15	male	60	NSCLC	IV	21	15	male	56	NSCLC	IV	21
16	female	48	NSCLC	IV	21	16	male	74	NSCLC	IV	21
17	female	52	NSCLC	IV	21	17	male	55	NSCLC	IV	21
18	female	57	NSCLC	IV	21	18	male	62	NSCLC	IV	21
19	male	63	NSCLC	IV	21	19	female	56	NSCLC	III	21
20	female	61	NSCLC	IV	21	20	male	58	NSCLC	IV	21
21	female	46	NSCLC	IV	21	21	male	53	NSCLC	IV	21
22	male	68	NSCLC	IV	21	22	male	52	NSCLC	IV	21
23	male	67	NSCLC	IV	21	23	male	40	NSCLC	IV	21
24	female	64	NSCLC	IV	21	24	female	50	NSCLC	IV	21
25	male	54	NSCLC	IV	21	25	male	58	NSCLC	IV	21
26	female	55	NSCLC	IV	21	26	male	72	NSCLC	IV	21
27	male	59	NSCLC	IV	21	27	female	43	NSCLC	IV	21
28	female	49	NSCLC	IV	21	28	female	74	NSCLC	IV	21
29	male	75	NSCLC	IV	21	29	male	66	NSCLC	IV	21
30	male	61	NSCLC	IV	21	30	female	63	NSCLC	IV	21
31	female	58	NSCLC	IV	21						
32	female	72	NSCLC	IV	21						
33	male	50	NSCLC	IV	21						
34	female	69	NSCLC	IV	21						
35	male	62	NSCLC	IV	21						
36	female	48	NSCLC	IV	21						
37	female	61	NSCLC	IV	21						
38	male	63	NSCLC	IV	21						
39	female	57	NSCLC	IV	21						
40	male	80	NSCLC	IV	21						
41	male	64	NSCLC	IV	21						
42	female	74	NSCLC	IV	21						
43	male	45	NSCLC	IV	21						
44	female	55	NSCLC	IV	21						
45	male	64	NSCLC	IV	21						
46	female	49	NSCLC	IV	21						
47	male	64	NSCLC	IV	21						
48	female	67	NSCLC	III	21						
49	male	49	NSCLC	IV	21						
50	male	69	NSCLC	IV	21						
51	female	63	NSCLC	IV	21						
52	male	27	NSCLC	IV	21						
53	male	83	NSCLC	IV	21						

For cytokines detection											
Treatment group						Randomized controlled trial					
Case ID	Gender	Age	Types of cancer	Stage at diagnosis	Docetaxel +Rocaltrol (day)	Case ID	Gender	Age	Types of cancer	Stage at diagnosis	Docetaxel without Rocaltrol (day)
1	male	57	NSCLC	III	21	1	male	64	NSCLC	III	21
2	female	64	NSCLC	IV	21	2	female	84	NSCLC	IV	21
3	male	71	NSCLC	II	21	3	female	27	NSCLC	IV	21
4	male	76	NSCLC	III	21	4	male	54	NSCLC	IV	21
5	male	64	NSCLC	III	21	5	male	60	NSCLC	III	21
6	female	59	NSCLC	IV	21	6	female	77	NSCLC	IV	21
7	female	56	NSCLC	IV	21	7	male	56	NSCLC	IV	21
8	female	71	NSCLC	IV	21	8	female	54	NSCLC	IV	21
9	male	62	NSCLC	IV	21	9	male	39	NSCLC	III	21
10	male	67	NSCLC	IV	21	10	female	66	NSCLC	IV	21
11	female	71	NSCLC	IV	21	11	male	69	NSCLC	III	21
12	male	36	NSCLC	IV	21	12	male	57	NSCLC	IV	21
13	male	61	NSCLC	III	21						
14	female	56	NSCLC	IV	21						
15	male	70	NSCLC	IV	21						
16	male	61	NSCLC	IV	21						
17	female	51	NSCLC	IV	21						

Table 3

Gene name	number	All oligos are listed in 5' to 3' sequence
VDR	sgRNA-1	ACGTTCCGGTCAAAGTCTCC
	sgRNA-2	GATGCGGCAGTCCCCGTTGA
	sgRNA-3	CTGCCGGCTCAAACGCTGTG

Table 4

Primers used for RT-qPCR	All primers are listed in 5' to 3' sequence	
Primer name	Forward Primer	Reverse Primer
human IFNG	TGAATGTCCAACGCAAAGCA	CTGGGATGCTCTTCGACCTC
human IL2	AACCTCAACTCCTGCCACAA	GCATCCTGGTGAGTTTGGGA
human IL4	AGCAGTTCCACAGGCACAAG	ACTCTGGTTGGCTTCCTTCAC
human IL6	TTCGGTCCAGTTGCCTTCTC	TGAGATGCCGTCGAGGATG
human IL10	GAGGAAAAAAAAATGTTCTTTGGGGA	GGGGCTCCCTGGTTTCTCTTCCTAA
human TGFβ	ATTCCTGGCGATACCTCAGC	CTCAACCACTGCCGCACAA
human VDR	GTGGACATCGGCATGATGAAG	GGTCGTAGGTCTTATGGTGGG
human Perforin	GGCTGGACGTGACTCCTAAG	CTGGGTGGAGGCGTTGAAG
human Fas	GGACCCTCCTACCTCTGGTT	ACCTGGAGGACAGGGCTTAT
human FasL	CTCCGAGAGTCTACCAGCCA	TGGACTTGCCTGTTAAATGGG
human Granzyme	CCCTGGGAAAACACTCACACA	GCACAACTCAATGGTACTGTCG
human TNFα	CACAGTGAAGTGCTGGCAAC	AGGAAGGCCTAAGGTCCACT
human GAPDH	TTCGACAGTCAGCGCATCTTCTT	GCCCAATACGACCAAATCCGTTGA
human β-actin	CATGTACGTTGCTATCCAGGC	CTCCTT AATGTCACGCACGAT

Table 5

Pyrosequencing	All primers are listed in 5' to 3' sequence	5' modification
1F	TTGGTAGTATAGTGGGTGTTG	NA
1R	CCCCACTCTAAAAAAAAAACATTAAAACAC	5'-Biotin
1S	TTATATAGTTTTATATTTTTGAGAT	NA
2F	TTGGTAGTATAGTGGGTGTTG	5'-Biotin
2R	AATAATAAAATACCTCAAACTCATCAACTT	NA
2S	CAAACTCATCAACTTAAAAT	NA

Table 6

ChIP-qPCR	All primers are listed in 5' to 3' sequence	
Primer name	Forward Primer	Reverse Primer
human ChIP-CD28-S1	CAGATCAGGAGGGAGGGACA	ACAAACCCCTAGTGTTGTTACC
human ChIP-CD28-S2	ACCCACATACACAAAACCCC	CGGCCAACTGCTCTCTTCTT
human ChIP-CD28-S3	GAGCATGAGACACCAAGGGG	ATTCTACGTGCAAGCAGCCA
human ChIP-CD28-S4	GCTAAATGCTCCAGAGGGCT	GATGGGGACAGGTTGTGTCA
human ChIP-CD28-S5	GCCCATCATGTAGTGACCGA	ACCACAAGGCATCCTGACTG
human ChIP-CD28-S6	GCTGGAACCCTAGCCCATC	AACTAAGCCAATTGGAAGACCT
human ChIP-CD28-S7	GTATCTTAACAAAAGTCCCTTGGAA	ACATAGTGAAACCCCATCTCAAT
human ChIP-PD-1-Ct-1	GAACCTGAGCCCAGAGGGG	GCCTCTTTCCATATCCCGCC
human ChIP-PD-1-Ct-2	GTTTCTAGCCTCGCTTCGGT	GCGTTTGTGAAATGGCTTGCT
human ChIP-PD-1-VDR	GAACTGTGGCCATGGTGTGA	GCACAGGTGACCCTACTGAA
human ChIP-Tim-3-Ct1	ATAGGTGGGAGAAATGGGGGT	GCTCAAGAAGCCTTGATCCCA
human ChIP-Tim-3-Ct2	GAACCAGCTCCCTACACACA	GAATTGCTACTGAGACCAGCTT
human ChIP-Tim-3-VDR	GAGCCTTGACCAAGTTCATGC	AGCATAAGCCCCCTAAGTGAC
human ChIP-TIGIT-Ct1	CCAACTCACAGCCCCAAAAG	TCCATCAGGTGCTGACTCAC
human ChIP-TIGIT-Ct2	GGGTCTGGGGTAGATTCCCT	CTTGCCAGCTAGACCCTGAG
human ChIP-TIGIT-VDR	CAACTGTGTTGAGAGCCTGC	GTGCCTACAAAGCACCAACC
human ChIP-CD28-Ct1	CAGATCAGGAGGGAGGGACA	ACAAACCCCTAGTGTTGTTACC
human ChIP-CD28-Ct2	GAGCATGAGACACCAAGGGG	ATTCTACGTGCAAGCAGCCA
human ChIP-CD28-VDR	ACCCACATACACAAAACCCC	CGGCCAACTGCTCTCTTCTT
