# Supplementary material for: 1α,25(OH)2D3 reverses exhaustion and enhances antitumor immunity of human cytotoxic T cells
Source: J Immunother Cancer. 2022 Mar 22;10(3):e003477. doi: 10.1136/jitc-2021-003477 (PMC8943781; doi:10.1136/jitc-2021-003477)
Supplement: Supplementary data [file jitc-2021-003477supp002.pdf]

Supplementary Figure Legends:

Figure S1. T cell exhaustion profile in patients with NSCLC. (**A** and **B**) Graphs showed the expression of PD-1^+, Tim-3^+, $TIGIT^+$ and $CD28^+$ on T cells ($CD8^+$ and Vγ9V$\delta2^+$ T) in healthy donors and patients with advanced lung cancer. *n*=24-48. Mann–Whitney test was used in (B) Healthy *vs* Patient group, PD-1^+, Tim-3^+, $TIGIT^+$ of $CD8^+$ (%), PD-1^+, Tim-3^+, $TIGIT^+$ of V$\delta2^+$ (%), V$\delta2^+$ of $CD3^+$ (%), $CD8^+$ of $CD3^+$ (%). Unpaired Student's *t*-test with Welch's correction were used in (B) Healthy *vs* Patient, $CD28^+$ of $CD8^+$ (%) and Tim-3^+ of V$\delta2^+$ (%). Unpaired Student's *t*-test was used in (B) Healthy *vs* Patient, $CD28^+$ of V$\delta2^+$ (%). $*P < 0.05$, $**P < 0.01$, $***P < 0.001$, $****P < 0.0001$, *n.s.*, not significant.

Figure S2. Vitamin D_3 level in NSCLC and healthy samples. (**A** and **B**) Graphs showed 25(OH)D_3 and 1α,25$(OH)_2D_3$ levels in sera of health donors and patients with NSCLC. *n*=12-29. Mann–Whitney test was used in (A and B). $*P < 0.05$, $**P < 0.01$, $***P < 0.001$, *n.s.*, not significant.

Figure S3. 25(OH)D_3 level is correlated with the expression of immune checkpoint receptors and CD28 expression. (**A** and **B**) Linear regression analysis between 25(OH)D_3 level and the expression of immune checkpoint receptors (PD-1^+, Tim-3^+, $TIGIT^+$, CTLA-4^+) and co-stimulatory molecule $CD28^+$ on T cells ($CD8^+$ and V$\delta2^+$ T cells) in patients with advanced lung cancer. *n*=23-26. The Pearson correlation was used in (A and B). $*P < 0.05$, $**P < 0.01$, $***P < 0.001$, *n.s.*, not significant.

Figure S4. 1α,25$(OH)_2D_3$ rescues T cells from exhaustion in patients with cancer. (**A**) Experimental designs for T cells stimulation in vitro. (**B-D**) Frequency of immune checkpoint receptors (PD-1^+, Tim-3^+, $TIGIT^+$) and $CD28^+$ on T cells ($CD8^+$ and Vγ9V$\delta2^+$ T cells) in PBMCs isolated from NSCLC donors

Li P, *et al. J Immunother Cancer* 2022; 10:e003477. doi: 10.1136/jitc-2021-003477

after 1α,25(OH)$_2$D$_3$ (50 nM) treatment for three times at 2-day intervals in vitro**.** *n*=12-22. Paired Student's *t*-test used in (C and D). *P < 0.05, **P < 0.01, ***P < 0.001, ****P <0.0001, *n.s.*, not significant.

Figure S5. 1α,25(OH)$_2$D$_3$ promotes T cell proliferation and reduces the expression of immune checkpoint receptors in healthy donors. (**A**) Gene expression of immune checkpoint receptors and costimulatory molecules regulated by 1α,25(OH)$_2$D$_3$ were determined by Quantitative Real-time PCR (CD3$^+$ cells were isolated from healthy donors). (**B-D**) Flow cytometry and Statistical analysis for the percentage of PD-1$^+$, Tim-3$^+$, TIGIT$^+$, and CD28$^+$ on T cells (CD4$^+$, CD8$^+$, and Vγ9Vδ2$^+$). Expression of PD-1$^+$, Tim-3$^+$, TIGIT$^+$ and CD28$^+$ on T cells (CD4$^+$, CD8$^+$ T and Vγ9Vδ2$^+$ T cells) in PBMCs of health donors, which were treated with 1α,25(OH)$_2$D$_3$ in vitro**.** *n*=6-13. **(E)** Co-expression of PD-1 with Tim-3 or TIGIT on T cells (CD4$^+$, CD8$^+$, and Vγ9Vδ2$^+$). *n*=4 healthy donors. (**F**) Dot plots showed frequency of Ki-67$^+$ and the expression of immune checkpoint receptors in Vγ9Vδ2$^+$ T cells. Paired Student's t-test (B-D). Unpaired Student's *t*-test was used in (E). *P < 0.05, **P < 0.01, ***P < 0.001, ****P <0.0001, *n.s.*, not significant.

Figure S6. Expression of immune checkpoint receptors and CD28 before and after treatment with calcitriol. (**A** and **B**) Comparison of PD-1$^+$, Tim-3$^+$, TIGIT$^+$, and CD28$^+$ levels on T cells (CD8$^+$, Vγ9Vδ2$^+$), in the peripheral blood of patients, before (baseline) and after (post) treatment with docetaxel and rocaltrol. Co-expression of PD-1 with Tim3 or TIGIT on T cells (CD8$^+$ and Vγ9Vδ2$^+$) derived from PBMCs of patients with NSCLC. (**C** and **D**) Patients with NSCLC received docetaxel treatment (75 mg/m^2). Blood samples were harvested at baseline and post-treatment (during docetaxel treatment). Paired Student's *t*-test used in (C and D). *P < 0.05, **P < 0.01, ***P < 0.001, ****P <0.0001, *n.s.*, not significant.

Figure S7. Docetaxel treatment alone does not affect T cell activation. (**A**) Overview of study design. Patients with NSCLC received one course of intravenous docetaxel (75 mg/m^2) without rocaltrol for 3 weeks. (**B**) Graphs showed the IFN-γ$^+$, TNF-α$^+$, Perforin$^+$, and Granzyme B$^+$ cells in CD4$^+$, CD8$^+$, and Vγ9Vδ2$^+$ T cells of NSCLC patients, before and after therapy with docetaxel (75 mg/m^2) for 3 weeks, followed by stimulation with anti-human CD3 and anti-human CD28 antibodies for 4 hours. (**C**) Graphs showed the level of IFN-γ, TNF-α, Perforin, and Granzyme B in serum of NSCLC patients, before (Pre) and after (Post) treatment with docetaxel (75 mg/m^2) and rocaltrol (0.5-2 μg/day) (n=17) or docetaxel alone (n=12) for 3 weeks. (**D**) The proportion of patients with increased or decreased cytokines, before (Pre) and after (Post) therapy with docetaxel (n=12) or docetaxel combined with rocaltrol (n=17). Patients with increase from baseline (pre) in the frequency of IFN-γ$^+$, TNF-α$^+$, Perforin$^+$, and Granzyme B$^+$ in CD4$^+$, CD8$^+$, and Vγ9Vδ2$^+$ T cells (post vs pre, fold change >1, increased). (**E**) The percentage of no recurrence patients, after treated with docetaxel combined with rocaltrol (patients with at least 1.2-fold increase from baseline in the frequency of TNF-α$^+$ or IFN-γ$^+$ on CD3$^+$ T cells). n=17. (**F**) The level of 1α,25$(OH)_2D_3$ in serum from patients with increased TNF-α$^+$ (post vs pre, fold change < 1.2, n=9; > 1.2, n=8) or IFN-γ$^+$ (fold change < 1.2, n=8; >1.2, n=9) CD3$^+$ T cells after treatment with docetaxel combined with rocaltrol. (**G**) Linear regression analysis between 1α,25$(OH)_2D_3$ level *v.s.* PD-1$^+$ on CD8$^+$ T cells derived from PBMCs of patients received docetaxel and rocaltrol (n=17). Unpaired Student's t-test (C and F). Pearson's correlations (G) Significance was set to P < 0.05 and represented as *P < 0.05, **P < 0.01, ***P < 0.001, ****P <0.0001, *n.s.*, not significant.

Figure S8. 1α,25$(OH)_2D_3$ could effectively down-regulate the expression of immune checkpoint receptors, and up-regulate that of CD28. (**A**) Vγ9Vδ2$^+$ T

cells were treated two times at 1-day intervals with indicated concentration of vitamin D (VD3, 25(OH)D_3, 1α,25$(OH)_2D_3$) in fresh medium. The frequency of PD-1$^+$, Tim-3$^+$, TIGIT$^+$, and CD28$^+$ on cell surface was detected by flow cytometry. (**B**) Human CD8$^+$ T cells were treated with vehicle or 1α,25$(OH)_2D_3$ (0, 0.5, and 10 nM) for 4 times at 1-day intervals, determined by flow cytometry.

Figure S9. 1α,25$(OH)_2D_3$ and VDR regulated the expression of immune checkpoint receptors. (**A** and **B**) VDR knockout CD8$^+$ T cells were generated by CRISPR-Cas9 technology. Knockout efficiencies were confirmed by real time PCR and immunoblotting. (**C**) Effects of *VDR* knockout on expression of PD-1, Tim-3, TIGIT, and CD28 on CD8$^+$ T cells, which were transduced with constructs containing *VDR* knockout (CRISPR-Cas9-VDR) or control vector (CRISPR-Cas9-NC), and were selected by puromycin. (**D**) The expression of PD-1 Tim-3, TIGIT, and CD28 on CD8$^+$ T cells with VDR overexpression were shown. Cells were stimulated with 1α,25$(OH)_2D_3$ for two times at 1-day intervals. (**E**) JASPAR website predicated VDR binding sites in the promoter regions of *Pdcd1*, *Tim-3*, *Tigit*, *Cd28* genes. Unpaired Student's *t*-test was used in (A). *P < 0.05, **P < 0.01, ***P < 0.001, ****P <0.0001, *n.s.*, not significant.

Figure S10. The combination of 5′-aza-2′-deoxycytidine or Trichostatin and 1α,25$(OH)_2D_3$ affected the expression of Tim-3 and TIGIT. (**A-D**) Representative flow cytometry profiles. The percentage of PD-1$^+$, Tim-3$^+$, TIGIT$^+$, and CD28$^+$ on T cells (Vγ9Vδ2$^+$, CD8$^+$) after treatment with 5′-aza-2′-deoxycytidine, 1α,25$(OH)_2D_3$, or the combination. Cells (Vγ9Vδ2$^+$, CD8$^+$ T) were treated with 5′-aza-2′-deoxycytidine (5-Aza-dC, 1 μM), Trichostatin (TSA, 100 nM), 1α,25$(OH)_2D_3$ (50 nM), or the combination for 48 hours. (**E** and **F**) Statistical analysis for the percentage of Tim-3$^+$ and TIGIT$^+$ on T cells (CD8$^+$, Vγ9Vδ2$^+$) were shown. *n*=4-5 healthy donors. Data represent mean±SD.

Figure S11. The cytokine production of T cells pretreated with 1α,25(OH)$_2$D$_3$ are associated with TCR activation. $CD8^+$ and Vγ9Vδ2^+ T cells were stimulated with anti-human CD3 and anti-human CD28 antibodies for 4 hours. (**A**) The percentage of T cells ($CD8^+$, Vγ9Vδ2^+) producing TNF-α and IFN-γ were shown. n=3 healthy donors. (**B-E**) Flow cytometry and statistical analysis were performed for the percentage of CD107a$^+$ (B), Fas$^+$ (C), NKG2D$^+$ (D), and Perforin$^+$ (E) on Vγ9Vδ2^+ T cells. n=3 healthy donors. Data represent mean±SD. Results in (B-E) are representative blots from 2 to 3 independent experiments. Unpaired Student's *t*-test (A-F). *$P < 0.05$, **$P < 0.01$, ***$P < 0.001$, ****$P < 0.0001$, *n.s.*, not significant.

Figure S12. The cytokine production of T cells pretreated with 1α,25(OH)$_2$D$_3$ was not affected by PMA plus Ion activation. (**A** and **B**) $CD8^+$ T and Vγ9Vδ2^+ T cells were stimulated with PMA and Ion for 4 hours. The percentages of T cells producing TNF-α or IFN-γ out of total T cells, pretreated with 1α,25(OH)$_2$D$_3$ or vehicle, were shown. Data represent mean±SD. Data are representative of three independent experiments. Unpaired Student's *t*-test (B). *$P < 0.05$, **$P < 0.01$, ***$P < 0.001$, ****$P < 0.0001$, *n.s.*, not significant.

Figure S13. Ca^{2+} affects the cytokine production of Vγ9Vδ2 T cells. (**A** and **B**) Intracellular Ca^{2+} was chelated with BAPTA-AM, followed by stimulation with anti-human CD3 and CD28 antibodies for 4 hours. TNF-α and IFN-γ production of Vγ9Vδ2 T cells were detected by flow cytometry. The percentage of TNF-α^+ and IFN-γ^+ Vγ9Vδ2 T cells were shown. Data represent mean±SD. Unpaired Student's *t*-test was used in (B). *$P < 0.05$, **$P < 0.01$, ***$P < 0.001$, ****$P < 0.0001$, *n.s.*, not significant.

Figure S14. Treatment with 1α,25(OH)$_2$D$_3$ promoted TNF-α production of Vγ9Vδ2 T cells under MCF-7 stimulation. (**A**) Overview of study design. **(B)** Vγ9Vδ2 T cells (vehicle or 1.25D$_3$ pretreated) were co-incubated with MCF-7 tumor cells (target) at different effector: target (E:T) ratios (5:1, 10:1) in the presence of Golgi Stop. The percentage of Vγ9Vδ2 T cells producing IFN-γ or TNF-α out of total Vγ9Vδ2 T cells were shown (*n*=6). Data are representative of three independent experiments. Data represent mean±SD. Unpaired Student's *t*-test (B). **P* < 0.05, ***P* < 0.01, ****P* < 0.001, *****P* <0.0001, *n.s.*, not significant.

Figure S15. Anti-tumor efficacy of γδ T cells was not promoted by addition of anti-PD-L1 antibody. (**A** and **B**) Experimental designs for MCF-7 and U2932 tumor models transferred with 1α,25(OH)$_2$D$_3$ pretreated Vγ9Vδ2 T cells (1.25D$_3$-γδ T) or vehicle pretreated Vγ9Vδ2 T cells (vehicle-γδ T). The control group mice were treated with an equivalent volume of PBS. In the group of anti-PD-L1 treatment combined with Vγ9Vδ2 T cells, mice were injected intravenously with anti-PD-L1 for every three days (three times in total) from day 7 after U2932 cells inoculation and tumor growth was recorded. *n*=6 mice per group. (**C** and **D**) Tumor growth kinetics (C), and survival curves (D) were shown. *n*=6 mice per group. (**E**) Tumor-infiltrating T cells were isolated and PD-1 expression was analyzed on day 18. *n*=6 mice. Data represent mean±SD. Two-way analysis of variance (C); log-rank (Mantel–Cox) test was used in (D). Unpaired Student's *t*-test (E). **P* < 0.05, ***P* < 0.01, ****P* < 0.001, *****P* <0.0001, *n.s.*, not significant.

Li P, *et al. J Immunother Cancer* 2022; 10:e003477. doi: 10.1136/jitc-2021-003477
