# Supplementary figures and images for: 1α,25(OH)2D3 reverses exhaustion and enhances antitumor immunity of human cytotoxic T cells
Source: J Immunother Cancer. 2022 Mar 22;10(3):e003477. doi: 10.1136/jitc-2021-003477 (PMC8943781; doi:10.1136/jitc-2021-003477)

Supplementary Figure S1

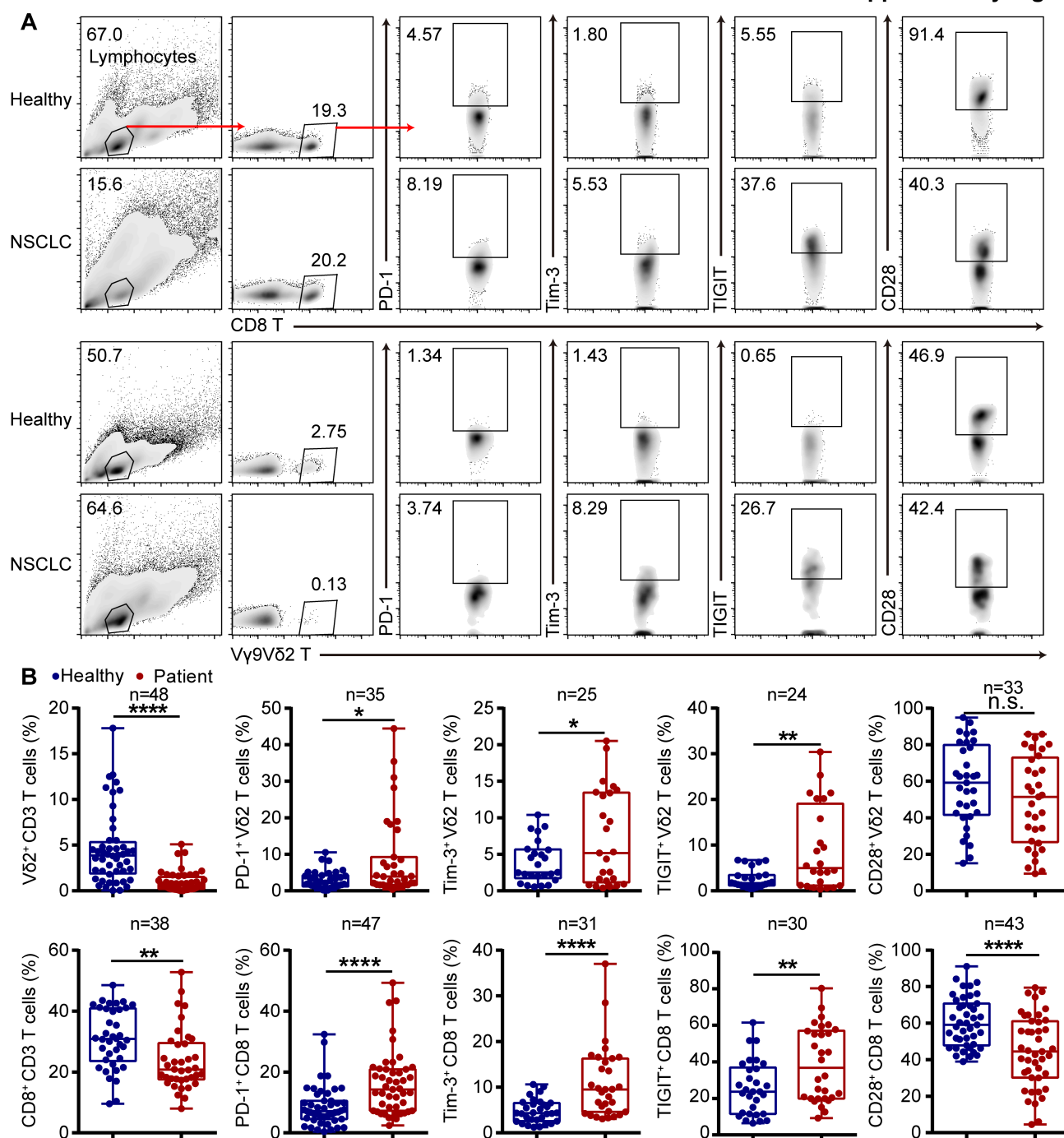

Supplement: Supplementary data [file jitc-2021-003477supp003.pdf]

Supplementary Figure S2

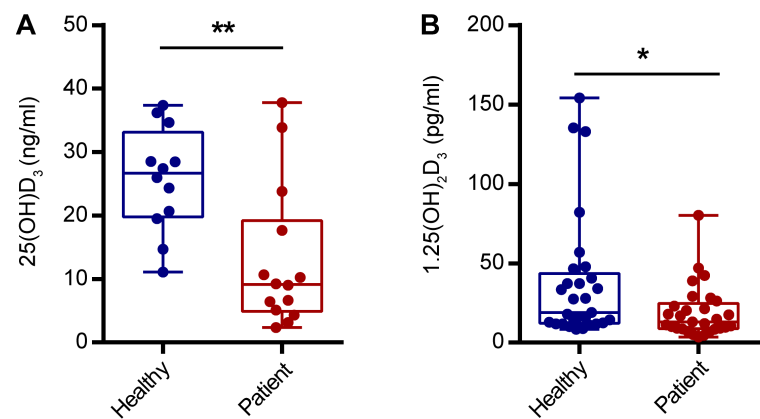

Supplement: Supplementary data [file jitc-2021-003477supp004.pdf]

Supplementary Figure S3

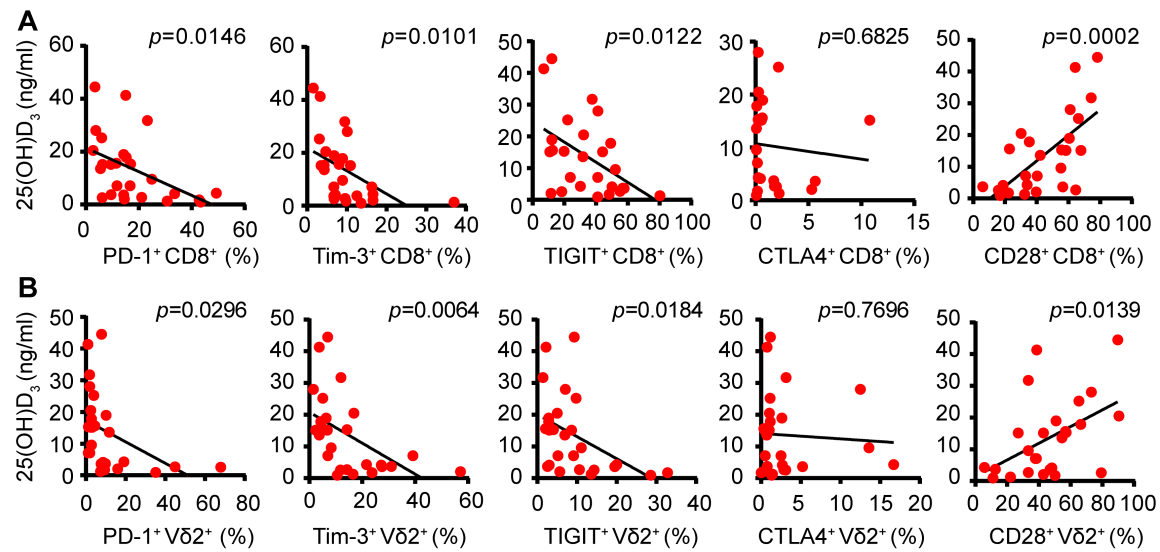

Supplement: Supplementary data [file jitc-2021-003477supp005.pdf]

## Supplementary Figure S4

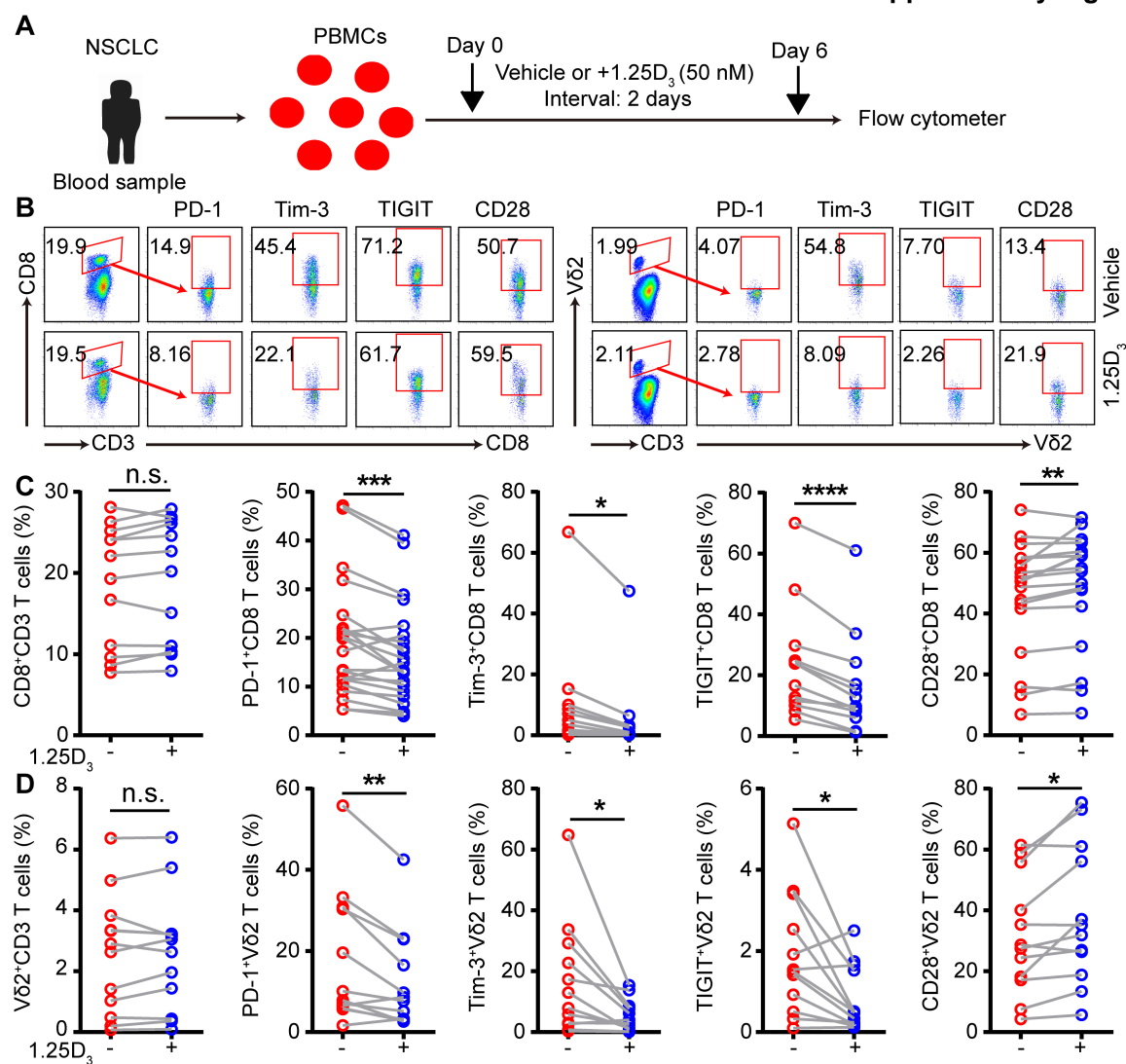

Supplement: Supplementary data [file jitc-2021-003477supp006.pdf]

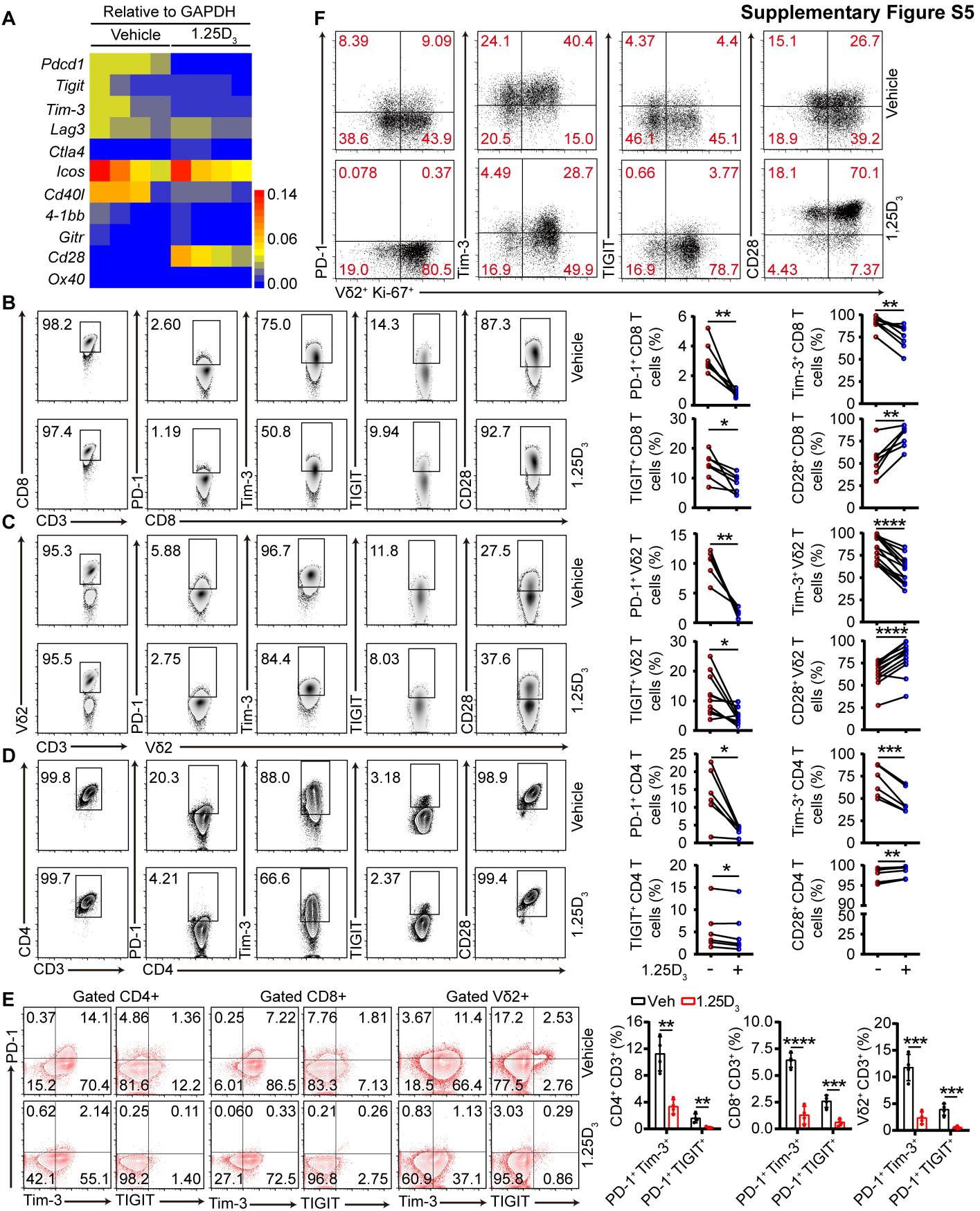

Supplement: Supplementary data [file jitc-2021-003477supp007.pdf]

Supplementary Figure S6

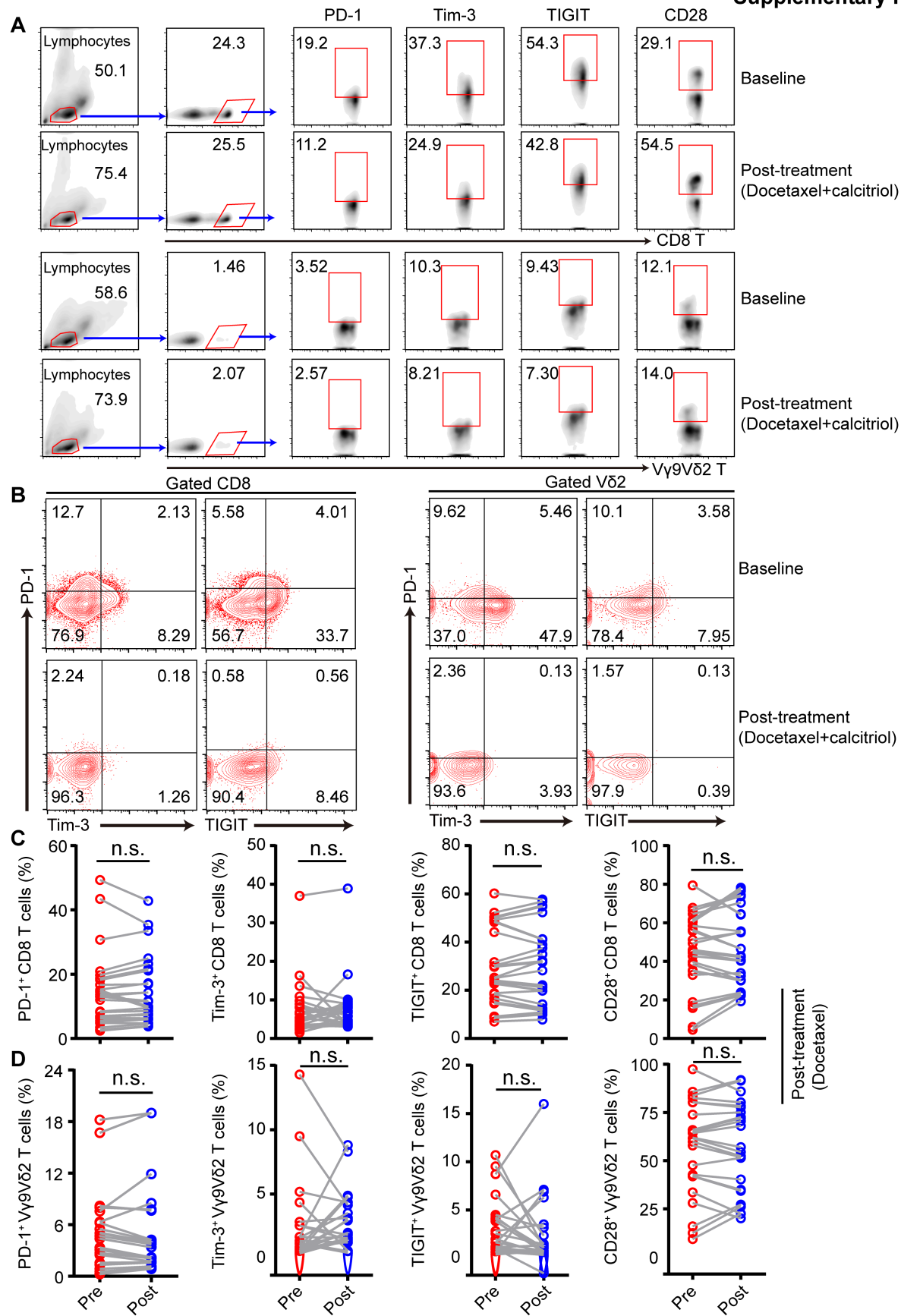

Supplement: Supplementary data [file jitc-2021-003477supp008.pdf]

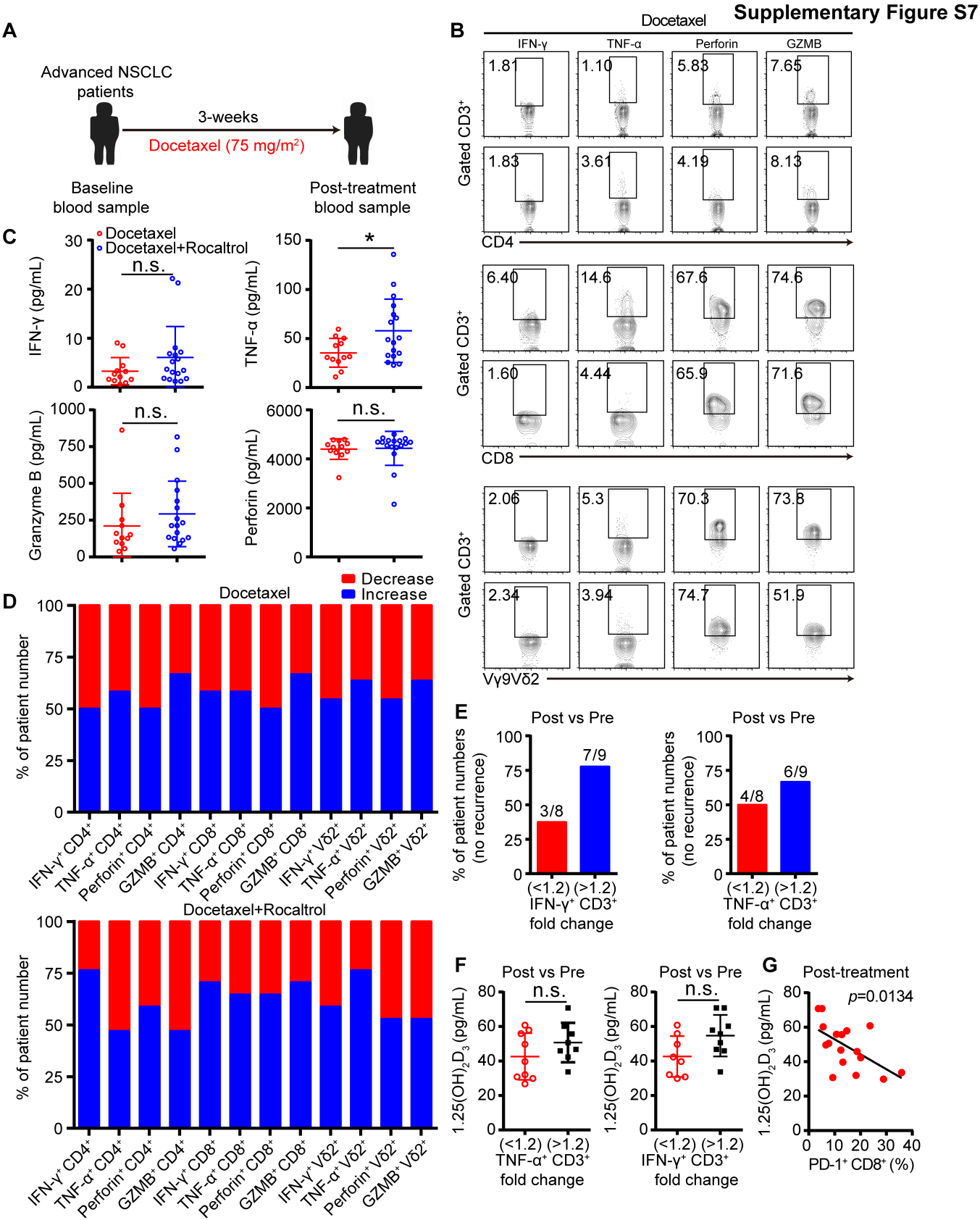

Supplement: Supplementary data [file jitc-2021-003477supp009.pdf]

Supplementary Figure S8

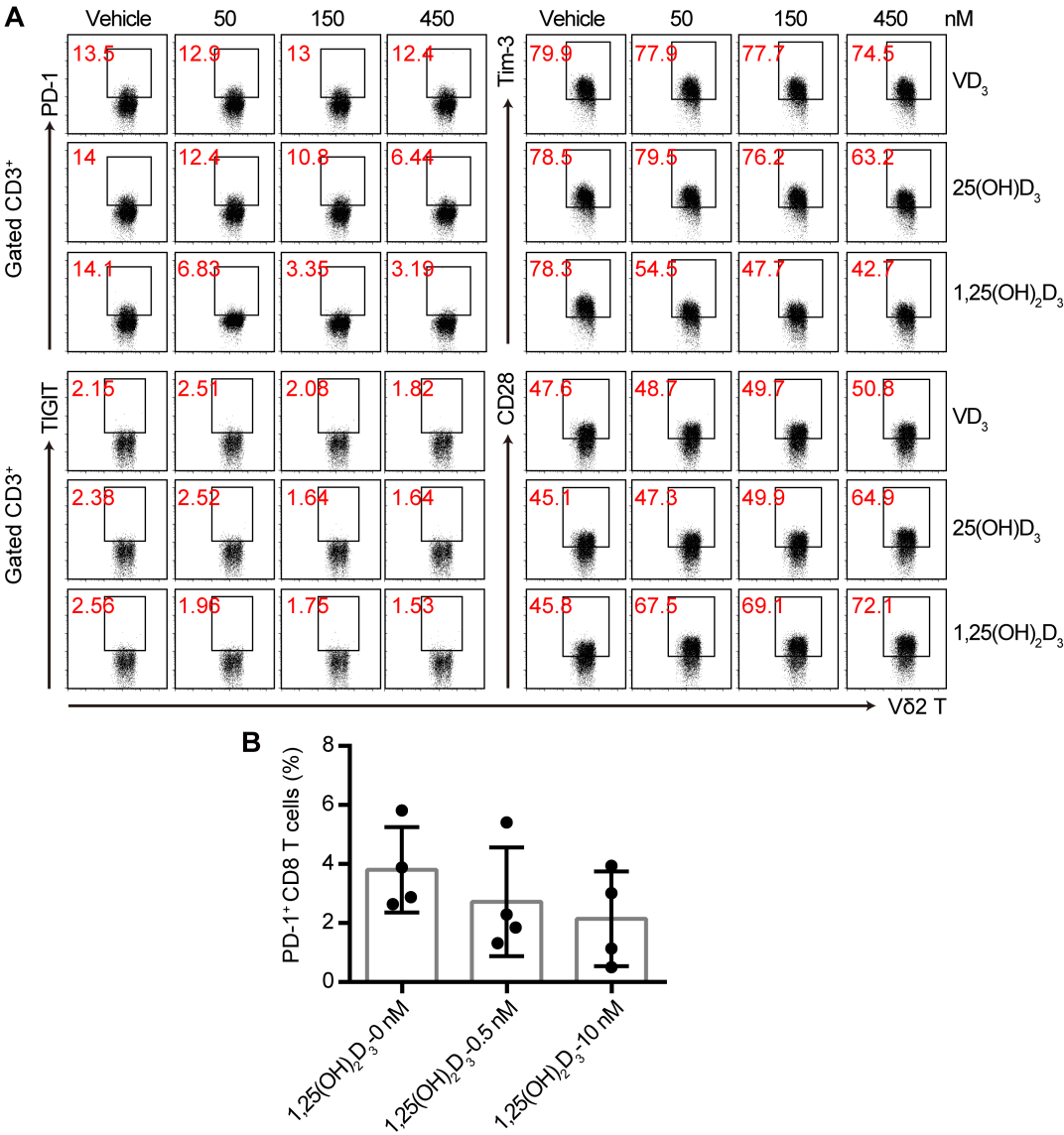

Supplement: Supplementary data [file jitc-2021-003477supp010.pdf]

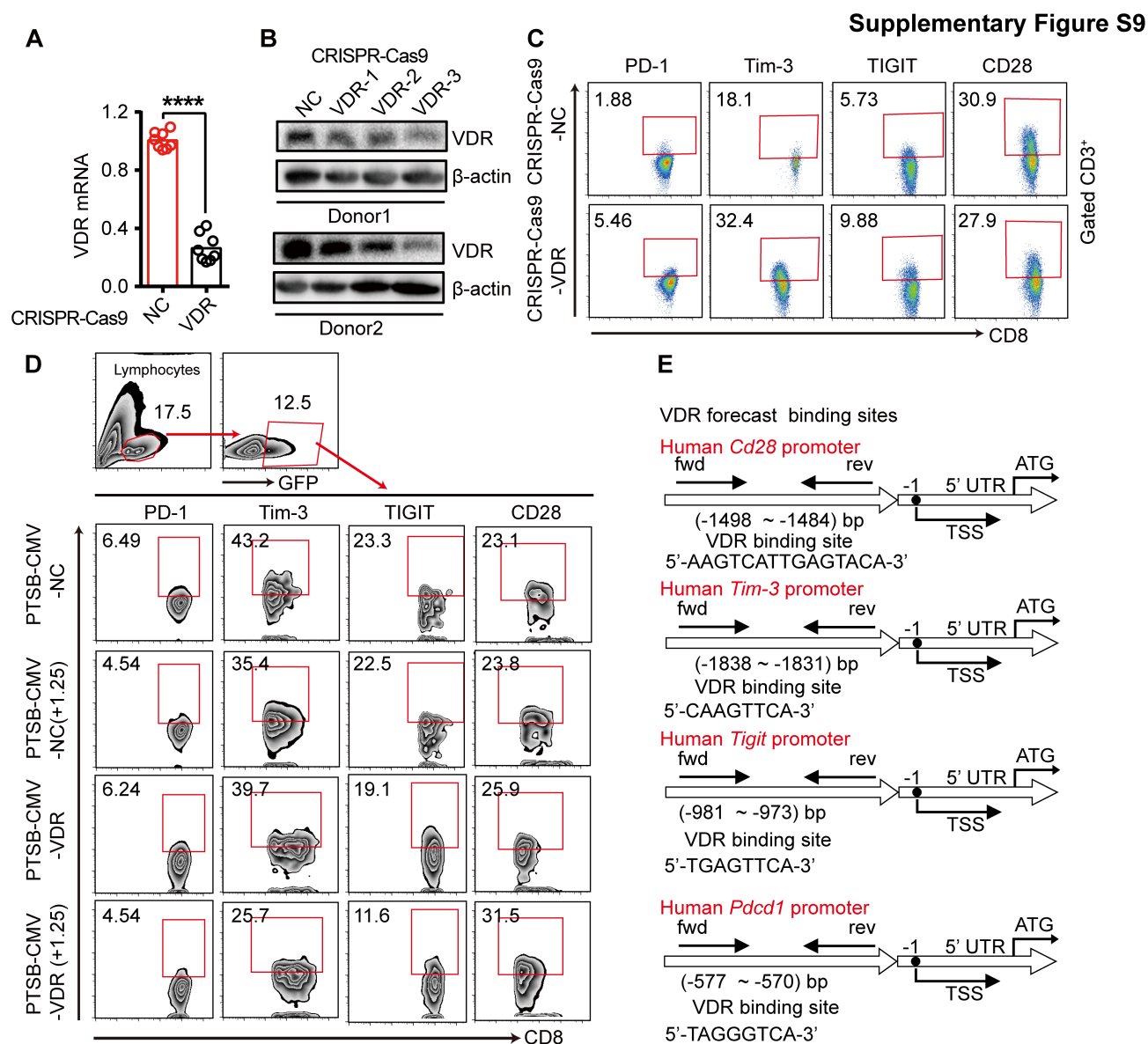

Supplement: Supplementary data [file jitc-2021-003477supp011.pdf]

Supplementary Figure S10

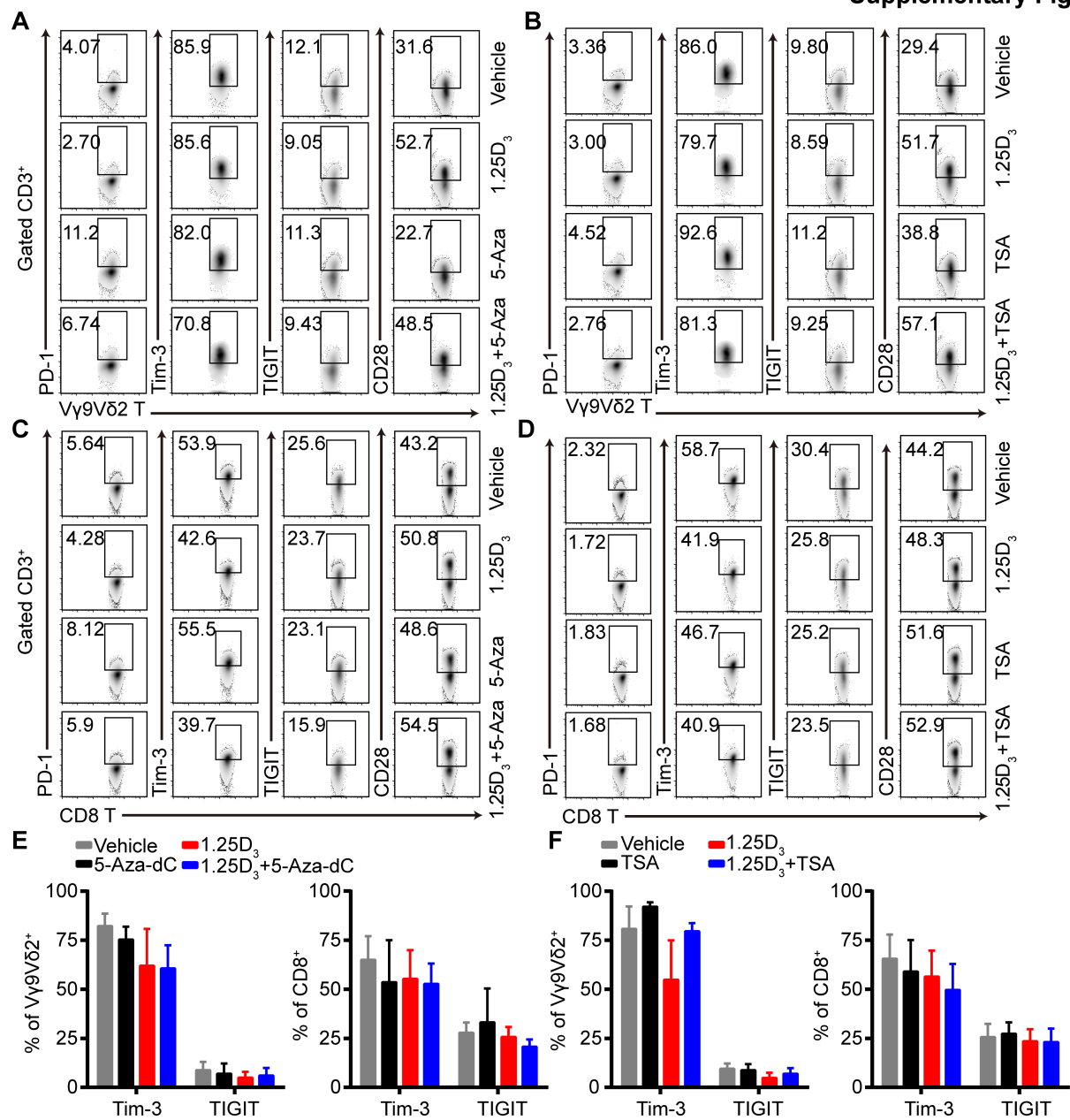

Supplement: Supplementary data [file jitc-2021-003477supp012.pdf]

## Supplementary Figure S11

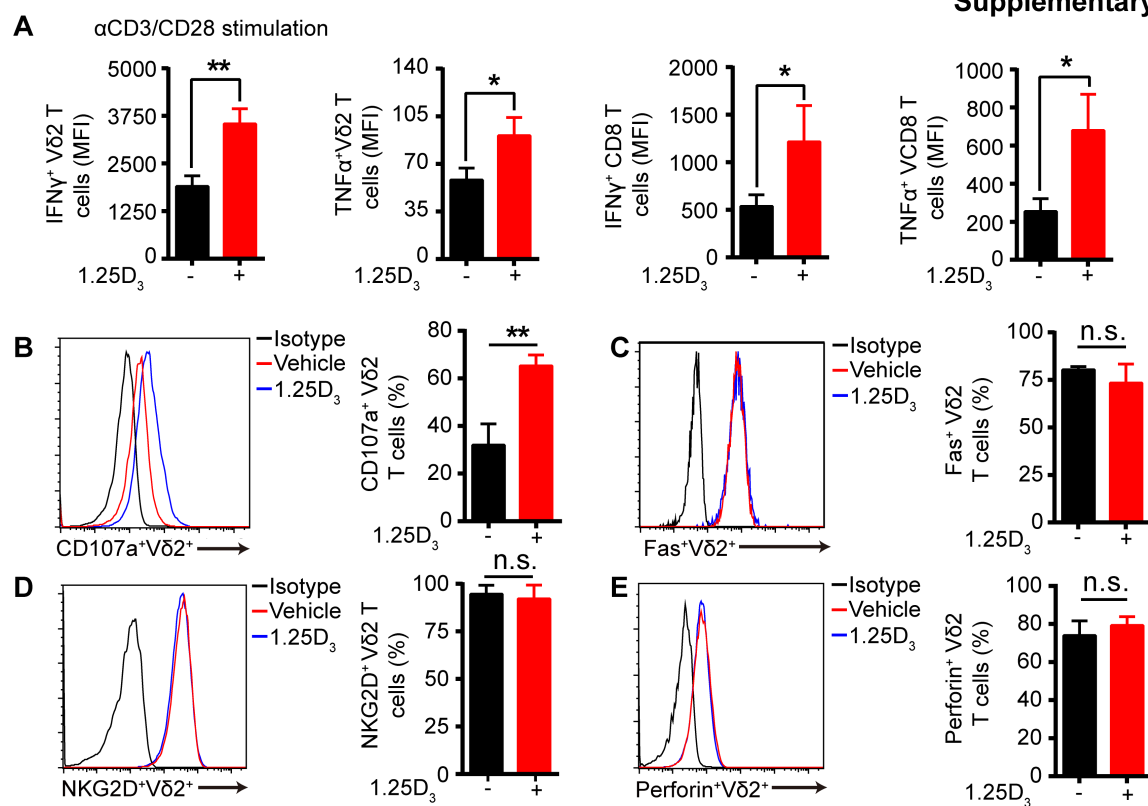

Supplement: Supplementary data [file jitc-2021-003477supp013.pdf]

Supplementary Figure S12

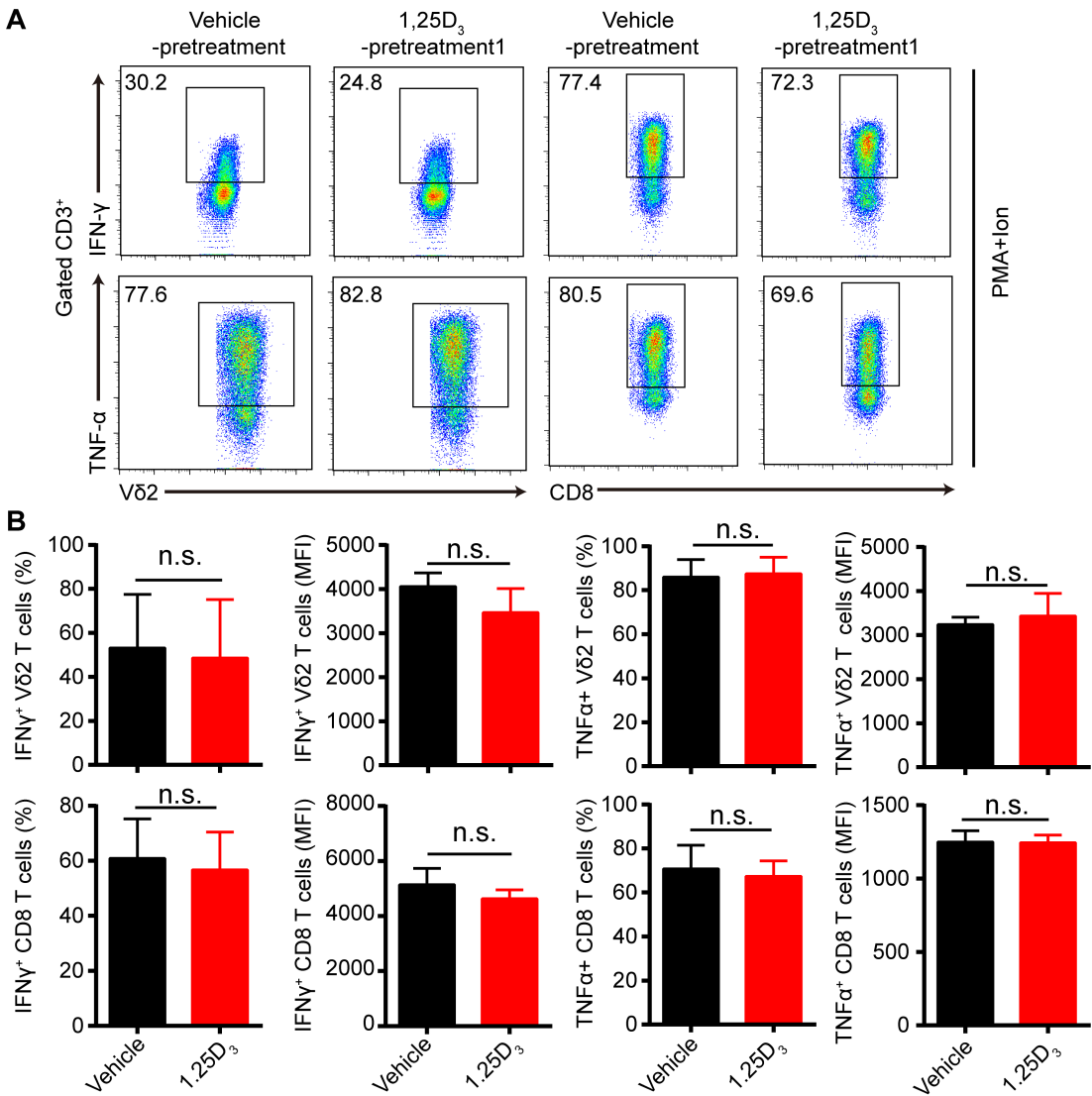

Supplement: Supplementary data [file jitc-2021-003477supp014.pdf]

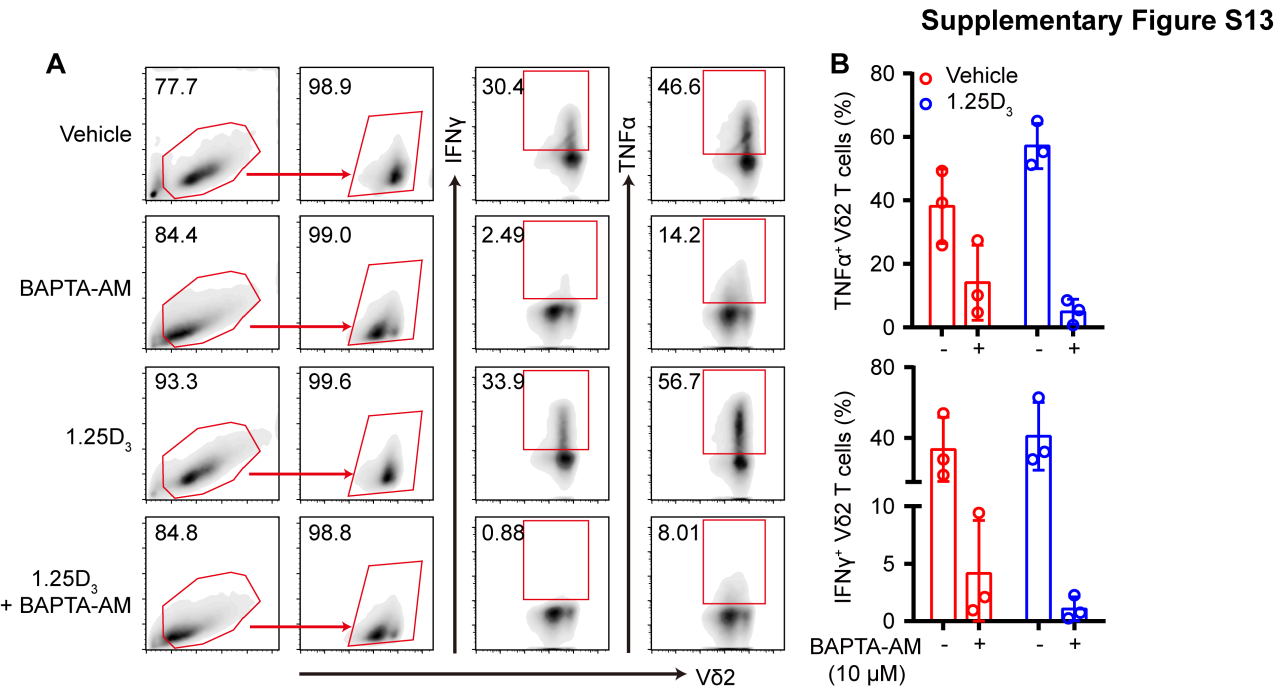

Supplement: Supplementary data [file jitc-2021-003477supp015.pdf]

Supplementary Figure S15

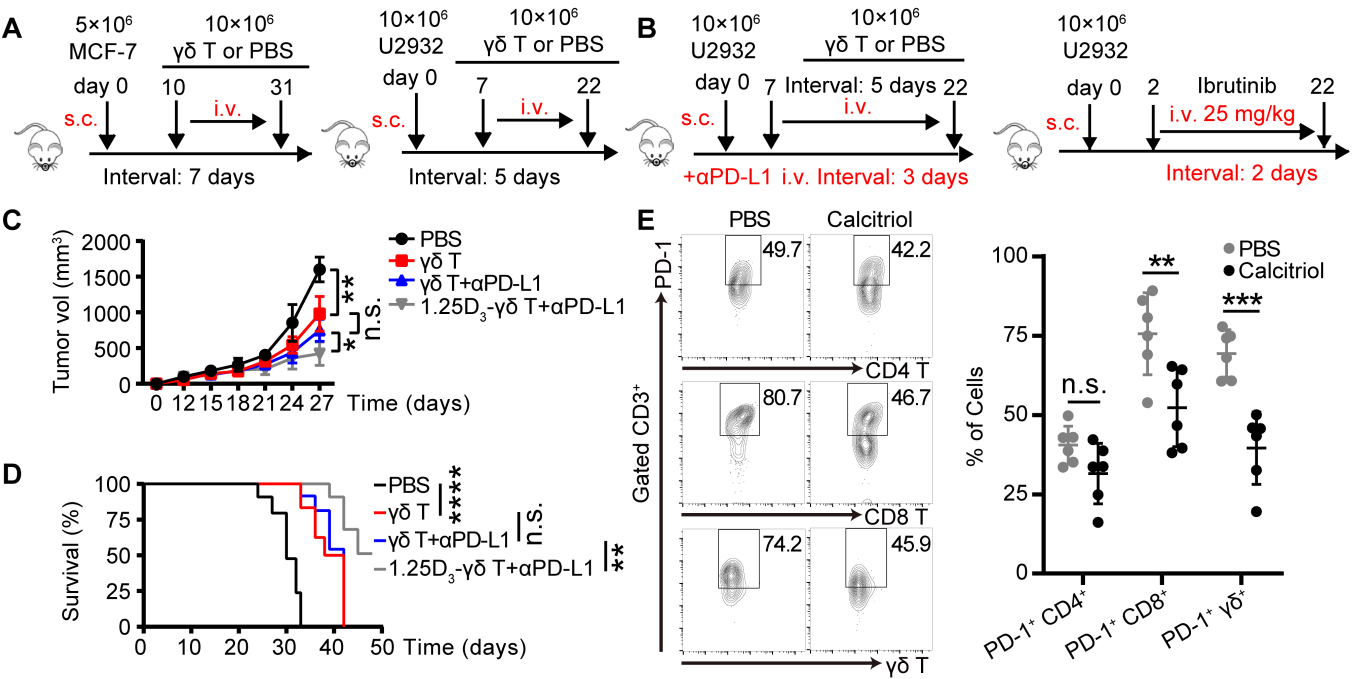

Supplement: Supplementary data [file jitc-2021-003477supp017.pdf]

## Graphical Abstract

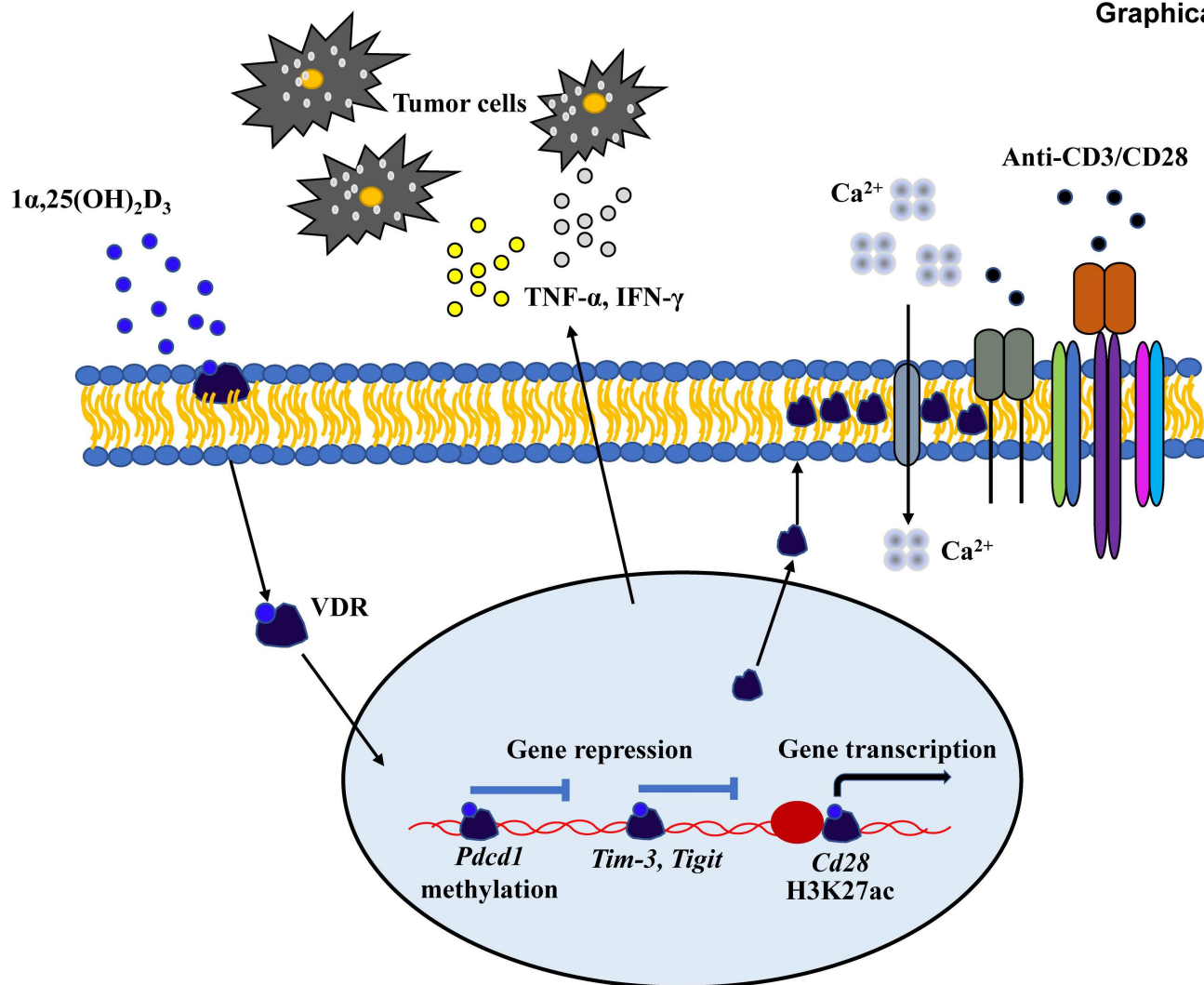

Supplement: Supplementary data [file jitc-2021-003477supp018.pdf]
