# Supplementary material for: 1α,25(OH)2D3 reverses exhaustion and enhances antitumor immunity of human cytotoxic T cells
Source: J Immunother Cancer. 2022 Mar 22;10(3):e003477. doi: 10.1136/jitc-2021-003477 (PMC8943781; doi:10.1136/jitc-2021-003477)

Supplementary Figure S14

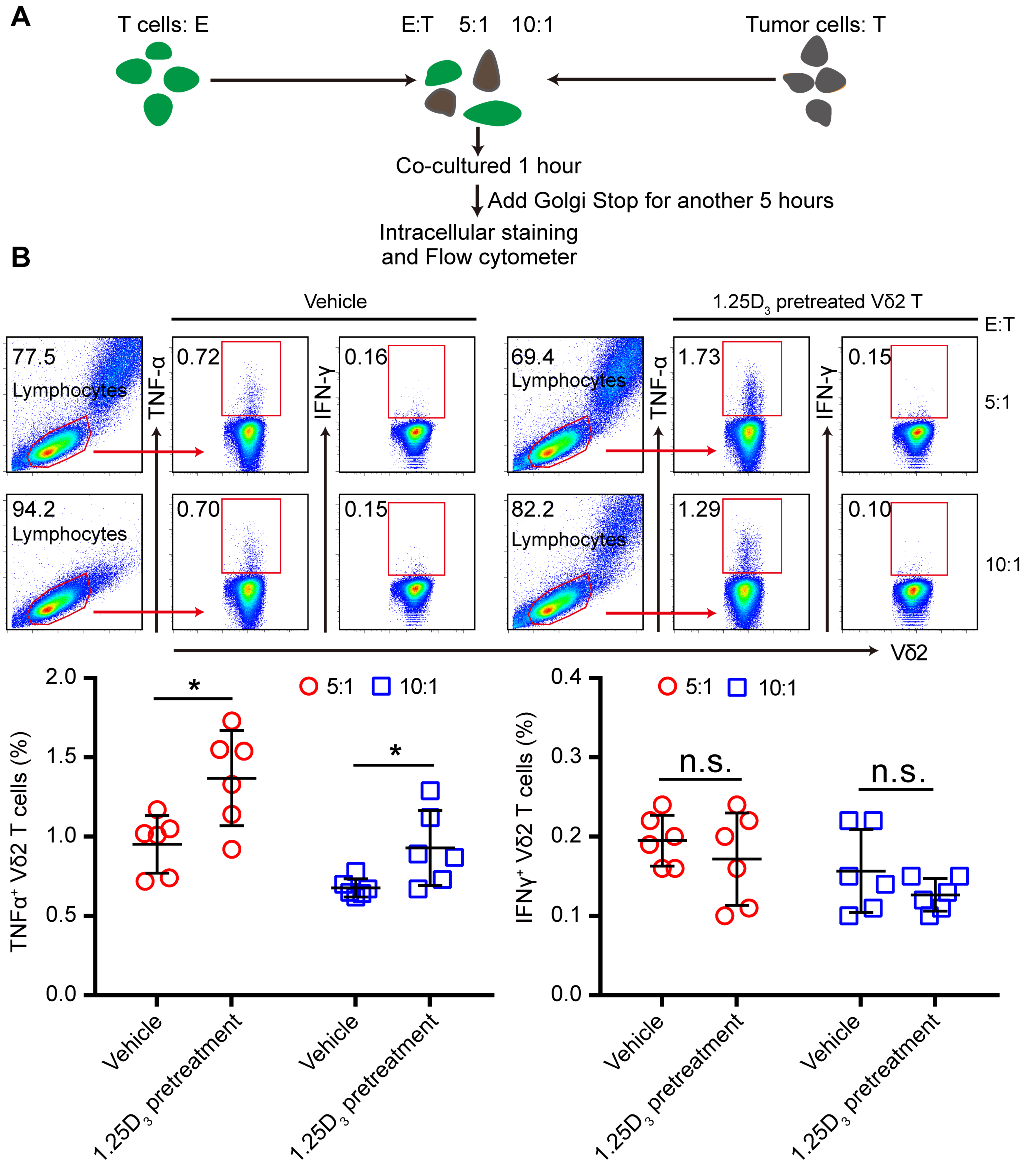

Supplement: Supplementary data [file jitc-2021-003477supp016.pdf]
